# Fermented mulberry (*Morus alba*) leaves suppress high fat diet-induced hepatic steatosis through amelioration of the inflammatory response and autophagy pathway

**DOI:** 10.1186/s12906-020-03076-2

**Published:** 2020-09-18

**Authors:** Mi Rim Lee, Ji Eun Kim, Ji Won Park, Mi Ju Kang, Hyeon Jun Choi, Su Ji Bae, Young Whan Choi, Kyung Mi Kim, Jin Tae Hong, Dae Youn Hwang

**Affiliations:** 1 Department of Biomaterials Science, College of Natural Resources & Life Science/Life and Industry Convergence Research Institute, Pusan National University, Miryang, 50463 South Korea; 2 Department of Horticultural Bioscience, College of Natural Resources & Life Science/Life and Industry Convergence Research Institue, Pusan National University, Miryang, 50463 South Korea; 3 Life Science Research Institute, Novarex Co., Ltd, Chungju, 28126 South Korea; 4 College of Pharmacy, Chungbuk National University, Chungju, 28644 South Korea

**Keywords:** Anti-hepatic steatosis, Mulberry leaves, Autophagy, Inflammation, Liver

## Abstract

**Background:**

A novel extract of mulberry leaves fermented with *Cordyceps militaris* (EMfC) is reported to exert anti-obesity activity, although their molecular mechanism during hepatic steatosis has not verified.

**Methods:**

To investigate the role of inflammation and autophagy during the anti-hepatic steatosis effects of EMfC, we measured alterations in the key parameters for inflammatory response and autophagy pathway in liver tissues of the high fat diet (HFD) treated C57BL/6N mice after exposure to EMfC for 12 weeks.

**Results:**

Significant anti-hepatic steatosis effects, including decreased number of lipid droplets and expression of Klf2 mRNA, were detected in the liver of the HFD + EMfC treated group. The levels of mast cell infiltration, expression of two inflammatory mediators (iNOS and COX-2), and the MAPK signaling pathway were remarkably decreased in the liver of HFD + EMfC treated group as compared to the HFD + Vehicle treated group. Furthermore, a similar inhibitory effect was measured for the expression levels of pro-inflammatory cytokines, including IL-1β, IL-6, TNF-α and NF-κB. The expression level of members in the AKT/mTOR signaling pathway (a central regulator in autophagy) was recovered after treatment with EMfC, and autophagy-related proteins (Beclin and LC3-II) were remarkably decreased in the HFD + EMfC treated group compared to the HFD + Vehicle treated group. Moreover, the HFD + EMfC treated group showed decreased transcript levels of autophagy-regulated genes including Atg4b, Atg5, Atg7 and Atg12.

**Conclusions:**

Taken together, findings of the present study provide novel evidences that the anti-hepatic steatosis of EMfC is tightly linked to the regulation of the inflammatory response and autophagy pathway in the liver tissue of HFD-induced obesity mice.

## Background

Inflammation is a physiological response generated against various physical, chemical and biological stimuli, in order to maintain homeostasis of the organism [1]. After initial stimulus from the external environment, this process is coordinated by many cell types including leucocytes and tissue-resident macrophages, as well as different inflammatory mediators such as cytokines, chemokines and vasoactive amines [2]. Hepatic inflammation is especially associated with obesity since activation of the inflammatory pathway is an outcome of hepatic steatosis and enhances the hepatic stress response. During obesity-induced liver inflammation, hypertrophic adipocytes secrete free fatty acid, while several immune cells (including Kupffer cells and hepatic stellate cells (HSC)) produce pro-inflammatory cytokines (TNF-α, IL-6, IL-1β and IL-10) and adipokines (leptin and adiponectin) [3, 4]. The increased fatty acid results in the secretion of inflammatory cytokines and ER stress through the activation of JNK [5, 6]. Simultaneously, excessive accumulation of free fatty acid in the liver inhibits autophagy at the autophagosome-lysosome fusion step through dysregulation of the ER calcium pump [7, 8].

Anti-inflammatory responses in hepatic steatosis have been widely investigated in the obesity model following exposure to various natural products including salmon cartilage proteoglycan [9], *Platycodon grandiflorum*-derived saponins (CKS) [10], *Acanthopanax koreanum* extract [11], large yellow tea [12], and *Emblica officinalis* [13]. However, limited similar outcomes for anti-obesity activity were observed in the analyses using extracts of fermented natural products. The extract of fermented *Moringa oleifera* decreases hepatic lipid accumulation, upregulates lipid metabolic genes, as well as decreases pro-inflammatory cytokine (IL-6 and IL-12) mRNA expressions in the liver of HFD-induced obesity model [14]. Similar effects were observed in the HFD-induced Wistar rats after treatment with fermented Fuzhuan tea. Increased mRNA levels of TNF-α observed in the adipose tissue of HFD animals were inhibited by Fuzhuan tea treatment [15]. Fermented soybean products also suppress the expression of NF-κB-induced inflammatory proteins, including COX-2, iNOS and VCAM-1, in kidneys of HFD-induced SD rats [16].

Meanwhile, non-alcoholic fatty liver disease (NAFLD) has been well defined as an accumulation condition of excessive fat in the liver tissue without another clear causes such as alcohol intake [17]. This disease contributes to various metabolic syndromes such as obesity, diabetes, insulin resistance, hyperlipidemia and hypertension [18]. Also, NAFLD classifies into simple steatosis and nonalcoholic steatohepatitis (NASH) depending on the determination of inflammation and liver cell damage. Of these two types, NASH with hepatic cellular injury and inflammation effectively progresses to fibrosis, cirrhosis and liver cancer [19]. Until now, various candidates including chemical compounds and natural products have been reported to have potential for the treatment of NASH. They included cytoprotective agents (ursodeoxycholic acid), antioxidants (vitamin E and betaine), antidiabetic agent (thiazolidinediones and troglitazone) and antihyperlipidemic agent (bezafibrate) [20–25]. However, many issues remain unresolved extending the spectrum and verifying the action mechanism of treatment drugs for NASH. To overcome these issues, natural products have been received a great attention as one of the important basic materials because they may effectively improve symptoms and severity of NAFLD without significant adverse side effects [26–28].

Furthermore, the anti-hepatic steatosis of natural products is tightly linked with the activation of autophagy. After exposure to several products, including bergamot polyphenol fraction (BPF), quercetin, metformin and *Eucommia ulmoides* leaf extract, the autophagy markers and their signaling pathway were remarkably recovered in the obesity model [29–32]. However, the EMfCs effects on the autophagy response has not been analyzed in the liver tissue of HFD-induced obesity model during anti-hepatic steatosis, although some evidences for their anti-obesity effects have been recently reported. In our previous studies, anti-obesity effects and action mechanism of EMfC have been investigated in adipocytes and obesity model. EMfC stimulated the lipolysis via the increase of cAMP concentration and free glycerol level in 3 T3-L1 cells and the primary adipocytes of Sprague Dawley (SD) rats [33]. Also, EMfC treatment for 12 weeks induce the significant decrease on the number of lipid droplets, peroxisome proliferator-activated receptor-γ (PPAR-γ) mRNA, adipocyte protein 2 (aP2) mRNA, FAS cell surface death receptor mRNA as well as the increase on the phosphorylation of perilipin and hormone-sensitive lipase, and the expression of adipose triglyceride lipase in the liver tissue of HFD-induced obese C57BL/6N mice [32]. A similar effect was observed on the amount of abdominal fat, the size of adipocytes, low-density lipoprotein (LDL) level, triglyceride level and total cholesterol (TC) level in HFD-induced obese C57BL/6N mice [32]. The anti-obesity effect of EMfCs in the liver tissue was reported to be linked to the regulation of ER stress and ER stress-induced apoptosis [34]. Therefore, above previous results about beneficial effects of EMfCs show the need for additional mechanism study related to anti-hepatic steatosis in HFD-induced obese C57BL/6N mice.

In this study, we investigated the regulatory mechanism of the inflammatory responses and autophagy during the anti-hepatic steatosis activity of EMfC in a C57BL/6N mice with HFD-induced obesity. The results of the present study show that the anti-hepatic steatosis activity of EMfC may closely related to suppression of the inflammatory response and recovery of autophagy regulation.

## Methods

### Preparation and fermentation of EMfCs

EMfCs was prepared using the materials and methods described in the previous study [20]. Dried samples of mulberry leaves harvested from plantations in the Sangju district of Korea and identified by Professor Young Whan Choi, one author of our study. These samples were deposited as voucher specimens (accession number Mul-PDRL-1) at the herbarium of the Department of Horticultural Bioscience at College of Natural Resources and Life Science in Pusan National University. The *C. militaris* and the silkworm pupae powder used for fermentation provided from Professor Sang Mong Lee of the Department of Life Science and Environmental Biochemistry and the Jeongeup Agriculture Cooperative Federations for Silkworm Farming (Jeongeup, Korea).

After sterilizing the mulberry powder at 121 °C for 60 min, these powders were mixed with 50% silkworm powder (SWP) and 10% *C. militaris* (v/w), and subsequently fermented in a shaking incubator (#SI-600R, Medline Scientific Co., Oxfordshire, UK) at 130 xg and 25 °C for 4 weeks. Also, the fermented mixture of mulberry powder was harvested from the glass flask and applied to extraction procedure in 95% EtOH. Extraction mixture containing the fermented mixture and the solvent (fixed ratio 1:10) was sonicated for 1 h using a JAC ultrasonic device (KODO, Hwangseong, Korea) and collected the supernatant after centrifugation at 13,000 xg for 20 min. This resultant supernatant was filtered, evaporated and lyophilized under previously reported conditions to prepare EMfCs [32]. Finally, EMfC were completely dissolved in dimethyl sulfoxide (DMSO)(#D2660, Sigma-Aldrich Co., St Louis, MO, USA) at a concentration of 50 mg/mL in 1% diluted DMSO solution based on previous studies showing non-toxic concentrations of DMSO to prevent animal toxicity [35].

### Experimental scheme for mice study

In vivo animal study was carried out as described in previous study using HFD-induced model. Experimental protocol for the obesity model was carefully reviewed based on ethical and scientific care guideline, and approved by the Pusan National University-Institutional Animal Care and Use Committee (PNU-IACUC; Approval Number PNU-2017-1519). Male C57BL/6N mice with eight weeks of age were obtained from Samtako BioKorea Inc. (Osan, Korea). All mice used study were provided with ad libitum access to water and an irradiated standard chow diet (Samtako BioKorea Co., Osan, Korea) containing crude protein (25.43%), moisture (12.5%), crude fat (6.06%), crude ash (5.31%), crude fiber (3.9%), calcium (1.14%) and phosphorus (0.99%). During all experimental period, they were breed in a specific pathogen-free (SPF) environmental condition under a strict light cycle (lights on at 08:00 A.M. and off at 08:00 P.M.) at 23 ± 2 °C temperature and 50 ± 10% relative humidity. Also, all mice were housed solid-bottom cage with wood shavings at the Pusan National University-Laboratory Animal Resources Center, which is accredited by the Korea Ministry of Food and Drug Safety (MFDS; Accredited Unit 000231) and Association for Assessment and Accreditation of Laboratory Animal Care (AAALAC) International (Accredited Unit 001525). The microbiological status of all mice have regularly monitored by animal health report that detected virus (4 species), bacteria and mycoplasma (7 species), and parasite (3 species).

Following acclimation for 1 week, the C57BL/6N mice (*n* = 28) were randomly divided into one of four experimental groups (7 mice/group): (1) NO treated group, (2) HFD + Vehicle (Olive oil + 1% DMSO) treated group, (3) HFD + OT treated group and (4) HFD + EMfC treated group. The animal number for each group was calculated based on the anti-obesity effects of mulberry leaves according to Ann et al. using G power 3.1 [36]. The No treated group fed a standard diet, while three HFD treated groups fed HFD containing 60% kcal fat (#D12492, Research Diets, Inc., New Brunswick, USA) for 12 weeks. HFD + EMfC treated group were orally administered 50 mg/kg body weight EMfC five times a week at 10 A.M., while HFD + OT and HFD + Vehicle treated group was received 10 mg/kg Orlistat (OT) or olive oil + 1% DMSO solution in the same way. But, No treated group did not receive any treatment during all experimental period. At 24 h after the final treatment, mice of subset groups were euthanized using CO_2_ gas in euthanasia chamber; tissue samples for analyses were subsequently collected and stored in Eppendorf tubes at − 70 °C. Five mice for liver analysis were selected from total mice of each subset group based on the increase rate of body weight and fat accumulation in the liver tissue.

### Measurement of organs weight, body weight and food efficiency

The weight of organs and body in all mice of No and HFD treated groups was measured by an electronic balance (Mettler Toledo, Greifensee, Switzerland) after the treatment of EMfC for 12 weeks. Food efficiency ratio was calculated using the following formula [37]:

$$ \mathrm{Food}\ \mathrm{efficiency}\ \mathrm{ratio}=\left(\mathrm{Body}\ \mathrm{weight}\ \mathrm{gain}\ \left(\mathrm{g}\right)/\mathrm{Food}\ \mathrm{intake}\ \left(\mathrm{g}\right)\right)\times 100 $$

### Histopathology for liver tissue

After collecting the liver tissues from mice of subset groups, these samples were fixed in 10% formalin solution for 48 h. The meddle region of second lob in the liver tissue were carefully trimmed from the fixed liver tissues, and then embedded into paraffin blocks. Following sectioning the tissue block into 4 μm thick slices, the liver sections were stained with hematoxylin and eosin (H&E) solution (Sigma-Aldrich Co.) and Masson’s trichrome (M&T) staining solution (Sigma-Aldrich Co.), and microscopically examined at 400× magnification for histopathological features. The pathological features and steatosis scoring of liver tissue were characterized by Professor Beum Seok Han, a pathologist, at the Department of Pharmaceutical Engineering, Hoseo University, Korea. In this histopathological scores system, scoring was evaluated based on the number of lipid droplet (1–3), fibrosis level (1–4) and inflammation (1–3) as described in previous study [38].

Meanwhile, mast cells infiltrated into liver tissue was stained with toluidine blue as described in previous study [39]. After deparaffinization and dehydration, liver sections on the slide glass were stained with 0.25% toluidine blue solution (Sigma-Aldrich Co.) and the presence of mast cells were observed by light microscopy. The number of cells per specific area was counted using the Leica Application Suite (Leica Microsystems, Wetzlar, Germany).

To stain oil red O, the liver tissue collected from HFD-induced obesity mice were directly embedded in OCT compound. This tissue blocks were frozen and sectioned into 10 μm thick slice. The liver sections on glass slides were fixed in 10% formalin solution for 10 min and subsequently treated with 60% isopropanol for 1 min, and then stained with Oil Red O solution (Sigma-Aldrich Co.) at 37 °C for 15 min. Following treatment of 60% isopropanol and dH_2_O, the liver tissues were counterstained with Mayer’s hematoxylin solution and observed by light microscopy.

### Quantitative real time (qRT)-PCR analysis

The frozen liver tissues of all mice were homogenized with Polytron PT-MR 3100 D Homogenizer (Kinematica AG, Lusern, Switzerland) in RNA Bee solution (Tet-Test Inc., Friendswood, TX, USA) based on manufacture’s protocol. After ethanol precipitation, total RNAs were harvested by centrifugation at 10,000 xg for 15 min, after which their concentration was determined by Nano-300 Micro-Spectrophotometer (Allsheng Instruments Co. Ltd., Hangzhou, China). The purity of total RNA was determined using the ratio of the absorbance at 260 and 280 nm. A value of 1.8–2.0 at an A260/A280 determined that the RNA has sufficient purity to be used in qRT-PCR analysis. Total complementary DNA (cDNA) against mRNA was synthesized using 200 units of Invitrogen Superscript II reverse transcriptase (Thermo Scientific, Wilmington, DE, USA). qRT-PCR was conducted with the cDNA template (1 μL), 2x Power SYBR Green (6 μL; Toyobo Life Science, Osaka, Japan), several following specific primers and appropriate buffer. The primer sequences to measure the expression level of target genes were as follows: IL-1β, sense 5′-GCA CAT CAA CAA GAG CTT CAG GCA G-3′ and anti-sense 5′-GCT GCT TGT GAG GTG CTG ATG TAC-3′; IL-6, sense 5′-TTG GGA CTG ATG TTG TTG ACA-3′ and anti-sense 5′-TCA TCG CTG TTG ATA CAA TCA GA-3′; TNF-α, sense 5′-CCT GTA GCC CAC GTC GTA GC-3′ and anti-sense 5′-TTG ACC TCA GCG CTG ACT TG-3′; NF-κB, sense 5′-TGA TGA CAT ACT CCC ACA AG-3′ and anti-sense 5′- CAA TAT CCC CAG ACC TAA C-3′; iNOS, sense 5′-CAC TTG GAG TTC ACC CAG T^− 3^′ and anti-sense 5′-ACC ACT CGT ACT TGG GAT GC-3′; COX-2, sense 5′-CAG GTC ATT GGT GGA GAG GTG TAT C^− 3^′ and anti-sense 5′-CCA GGA GGA TGG AGT TGT TGT AGA G-3′; Atg4b, sense 5′-CTA TGT GGA GAC GCT GAA GCA CTG TTT C-3′ and anti-sense 5′-CTC TCC AGT CTC TCT ACA TCA GAA GAG-3′; Atg5, sense 5′-CCA AGA GTC AGC TAT TTG ACG-3′ and anti-sense 5′-TCC AAG GAA GAG CTG AAC TTG-3′; Atg7, sense 5′-CCT TGC TCA AAC ACT ACA GTG-3′ and anti-sense 5′-TGC TAT GTG TCA CGT CTC TAG-3′; Atg12, sense 5′-CCA TCC AAG GAC TCA TTG AC-3′ and anti-sense 5′- TTG CAG TAA TGC AGG ACC AG-3′; Klf2, sense 5′-CTG GAA TTG AAC CAC AGA GGA CTG AC-3′ and anti-sense 5′-GTC ACA GTC TGG ACA CTG GAA AGG TTT G-3′; β-actin, sense 5′-TGG AAT CCT GTG GCA TCC ATG AAA C-3′ and anti-sense 5′-TAA AAC GCA GCT CAG TAA CAG TCC G-3′. PCR analyses were conducted at 95 °C for 15 s (denaturation), 64 °C for 30 s (annealing), and 72 °C for 60 s (extension) for 40 cycles. These reactions were run in StepOne™ Real-Time PCR System (Applied Biosystem, Poster city, CA, USA) and relative level of each gene was quantified based on a relative standard curve and comparative Ct (ΔΔCt) using StepOne Plus™ Systems software (Applied Biosystem,) as described in previous study [40].

### Western blot analysis

Total proteins from liver tissue (50 mg) were prepared using the Pro-Prep Protein Extraction Solution (Cat. No. 17081, Intron Biotechnology Inc., Seongnam, Korea). After the collection of proteins homogenates with centrifugation at 13,000 rpm for 5 min, the protein concentrations of each group were determined using a SMARTTM Bicinchoninic Acid Protein Assay Kit (Cat. No. 23225, Thermo Fisher Scientific Inc.). Total proteins (30 μg) were electrophoresed on 4–20% sodium dodecyl sulfate-polyacrylamide gel electrophoresis (SDS-PAGE) for 2 h, and they were subsequently transferred to nitrocellulose blotting membranes with a 0.45 μm pore size (Cat. No. 10600003, GE Healthcare, Little Chalfont, UK) for 2 h at 40 V. This membrane was incubated separately, overnight at 4 °C, with specific primary antibodies: anti-JNK antibody (#9252 s, Cell signaling Technology, Danvers, MA, US), anti-p-JNK antibody (#9251 s, Cell signaling Technology), anti-ERK antibody (#sc-94, Santa Cruz Biotechnology, Dallas, Texas, USA), anti-p-ERK antibody (#sc-7383, Santa Cruz Biotechnology), anti-p38 antibody (#9212 s, Cell Signaling Technology), anti-p-p38 antibody (#9211 s, Cell Signaling Technology), anti-Beclin antibody (#3738 s, Cell Signaling Technology), anti-LC3-I/II antibody (#3868 s, Cell Signaling Technology), anti-PI3K antibody (#4292 s, Cell Signaling Technology), anti-p-PI3K antibody (#4228 s, Cell Signaling Technology), anti-AKT antibody (#9272 s, Cell Signaling Technology), anti-p-AKT antibody (#4058 s, Cell Signaling Technology), anti-mTOR antibody (#2972 s, Cell Signaling Technology), anti-p-mTOR antibody (#2971 s, Cell Signaling Technology), and anti-β-actin antibody (#4967S, Cell Signaling Technology). After then, the probed membranes were then washed with washing buffer (137 mM NaCl, 2.7 mM KCl, 10 mM Na_2_HPO_4_, and 0.05% Tween 20), followed by incubation with 1:1000 diluted horseradish peroxidase (HRP)-conjugated goat anti-rabbit IgG (Cat. No. G21234, Invitrogen) at room temperature for 1 h. Each protein blotted membrane was developed using the Amersham™ ECL Select™ Western Blotting detection reagent (Cat. No. RPN2235, GE Healthcare). Finally, the chemiluminescence signals that derived from specific protein bands were measured using FluorChemi®FC2 (Alpha Innotech Co., San Leandro, CA, USA).

### Statistical analysis

The statistical significance between the groups was analyzed with One-way Analysis of Variance (ANOVA) (SPSS for Windows, Release 10.10, Standard Version, Chicago, IL, USA) followed by Tukey post hoc t-test for multiple comparisons. All values are presented as the means ± SD, and a *p* value (*p* < 0.05) is determined as statistically significant.

## Results

### Verification of anti-hepatic steatosis activity of EMfC

**Fig. 1:**
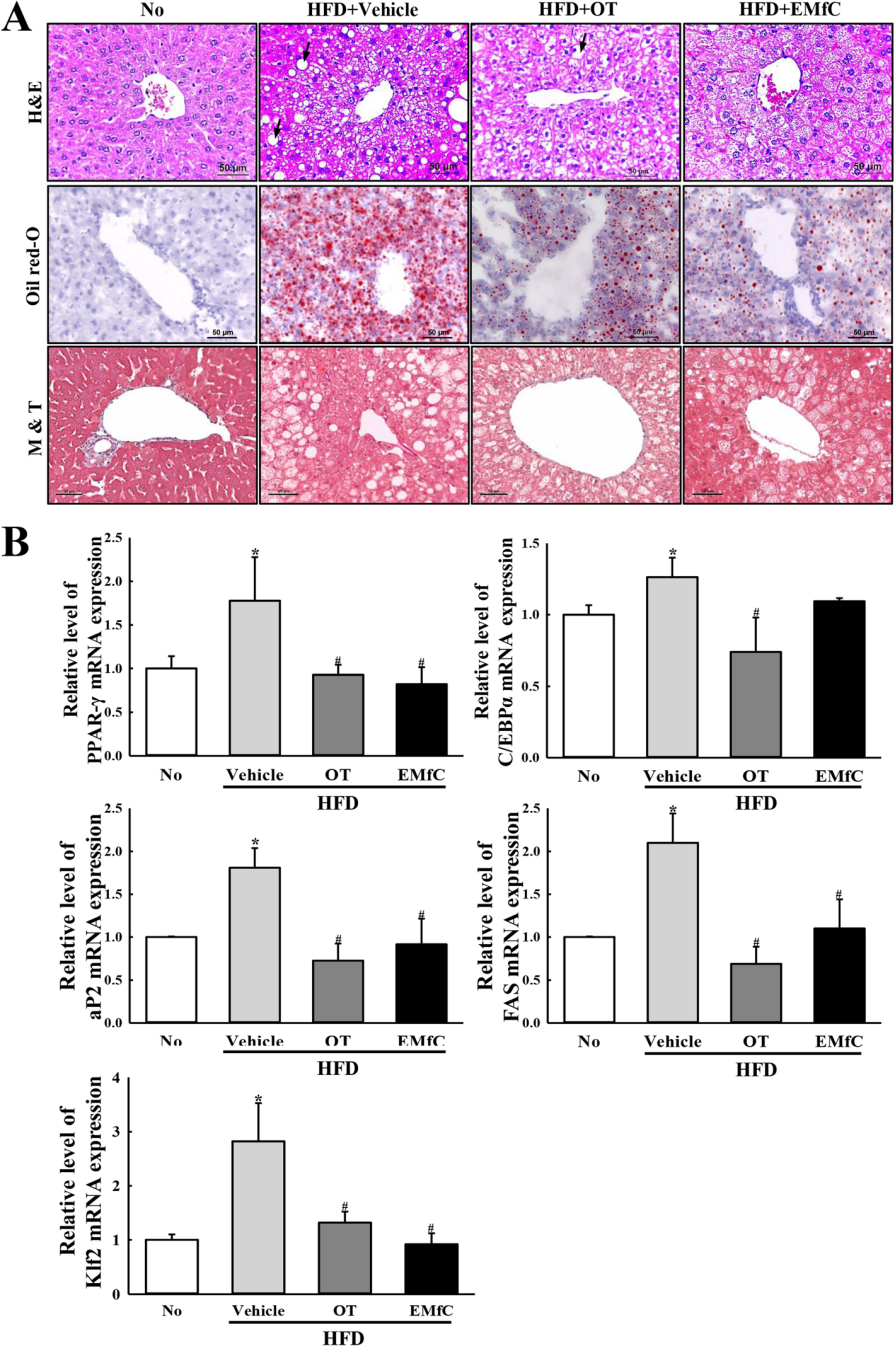
Fat accumulation on the liver histopathology. **a** After embedding and sectioning the fixed liver tissue of all mice, hematoxylin and eosin (H&E) and Masson’s trichrome (M&T) stained liver sections are shown at 400x magnification. Frozen sections of liver tissue were stained with Oil Red O to detect intracellular lipid droplets after fixation. Three to five mice per group were used for the preparation of the tissue sections, and H&E, M&T and Oil Red O staining was analyzed in duplicate in each liver tissue. **b** After exposure to EMfC (50 mg/kg) for 12 weeks, the levels of PPAR-γ, C/EBPα, FAS, aP2 and Klf2 mRNA in the total transcripts of liver were measured by qRT-PCR analyses using their specific primers. The relative levels of the PPAR, C/EBPα, FAS, aP2 and Klf2 mRNA were calculated based on the intensity of β-actin as an endogenous control. Three to five mice per group were used in the preparation of the total RNAs, and qRT-PCR analyses was assayed in duplicate for each sample. Data represents the mean ± SD. Asterisk symbol (*) indicates significance *P* < 0.05 as compared to the No treated group. Sharp symbol (#) indicates significant *P* < 0.05 compared to the HFD + Vehicle treated group

Firstly, we conformed the anti-hepatic steatosis activity of EMfC in liver tissue of HFD-induced obese mice through the regulation of weight gain as same as previous study (Table 1). In order to verify the inhibitory effect of EMfC on hepatic steatosis, the steatosis score based on lipid droplet number, fibrosis level and inflammation level were calculated in H&E, M&T and Oil Red O stained liver sections after exposure to EMfC. The number and size of lipid droplets were measured in H&E stained liver sections after exposure to EMfC. The HFD + Vehicle treated group had the number of lipid droplets and high average area of single droplet in the specific area. However, the levels were decreased in the HFD + EMfC and HFD + OT treated groups as compared to the HFD + Vehicle treated group, although the decrease rates differed. Also, the remarkable accumulation in lipid droplets was detected in Oil Red O stained liver tissue of HFD + EMfC and HFD + OT treated mice. A similar decrease was observed on the inflammation level after EMfC treatment, while the fibrosis level was constantly maintained in all experimental groups (Fig. 1a). Therefore, total score in HFD + Vehicle treated group was decreased in the HFD + EMfC treated groups (Table 2).

**Table 1:** Body weight and food efficiency in HFD-induced obese mice treated with EMfC

Category	No	HFD		
		Vehicle	OT	EMfC
Body weight (g)				
Initial	24.13 ± 1.86	24.91 ± 2.16	24.39 ± 1.99	24.16 ± 1.85
Final	27.14 ± 2.26	51.60 ± 1.95*	45.50 ± 2.76*^,#^	44.99 ± 1.47*^,#^
Change	3.01	26.69	21.11	20.83
Abdominal fat weight (g)	0.63 ± 0.22	3.32 ± 0.23*	3.05 ± 0.26*	3.18 ± 0.41*
Liver weight (g)	1.05 ± 0.13	2.91 ± 0.52*	1.94 ± 0.43*^,#^	2.04 ± 0.26*^,#^
Food efficiency ratio (%)	0.046 ± 0.019	0.227 ± 0.025*	0.167 ± 0.077*^,#^	0.150 ± 0.004*^,#^

Asterisk symbol (*) indicate significant *P* < 0.05 compared with the No treated group. Sharp symbol (#) indicates significant *P* < 0.05 compared with the HFD + Vehicle treated group

**Table 2:** Hepatic steatosis score in HFD-induced obese mice treated with EMfC

Category	No	HFD		
		Vehicle	OT	EMfC
Number of lipid droplet	0	3	1	1
Fibrosis level	0	0	0	0
Inflammation level	0	1	1	1
Total	0	4	2	2

After staining liver section with H&E, Oil red O and M&T solution, the histopathological scores was evaluated based on the number of lipid droplet (1–3), fibrosis level (1–4) and inflammation (1–3) as described in materials and methods

A similar effect was observed for levels of Klf2 (Krüppel-like Factor 2) transcript in the subset group; increased levels of Klf2 in the HFD + Vehicle treated group were remarkably recovered after exposure to EMfC (Fig. 1b). These results indicate that EMfC successfully inhibits hepatic steatosis in the liver tissue of HFD-induced obesity mice.

### Attenuation effects of EMfC on the regulation of HFD-induced inflammatory responses during anti-hepatic steatosis

**Fig. 2:**
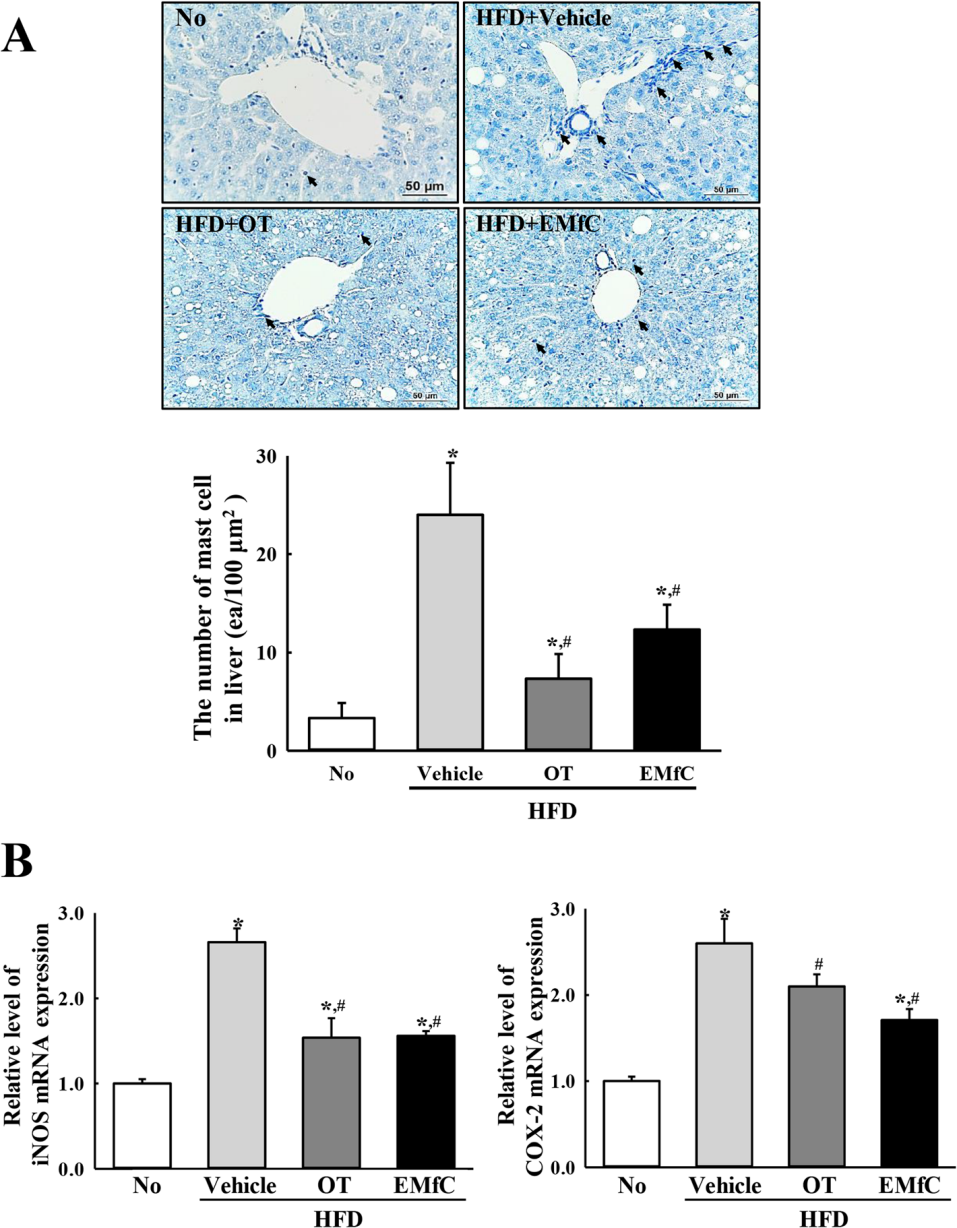
Infiltration of mast cells and expression levels of inflammatory mediators. **a** The infiltrated mast cells in the tissue sections of liver collected from HFD + EMfC treated mice were stained with toluidine blue, followed by observed and counted at 400x magnification. The arrow head in histological image indicates mast cells infiltrated into the liver tissue. Three to five mice per group were used for the preparation of the tissue sections, and toluidine blue staining and cell counting was analyzed in duplicate in each liver tissue. **b** The levels of iNOS and COX-2 mRNA in the total transcripts of liver were measured by qRT-PCR analyses using their specific primers. The relative levels of the iNOS and COX-2 mRNA were calculated based on the intensity of β-actin as an endogenous control. Three to five mice per group were used in the preparation of the total RNAs, and qRT-PCR analyses was assayed in duplicate for each sample. The values of data represent the mean ± SD. Asterisk symbol (*) indicates significant *P* < 0.05 compared to the No treated group. Sharp symbol (#) indicates significant *P* < 0.05 compared to the HFD + Vehicle treated group

**Fig. 3:**
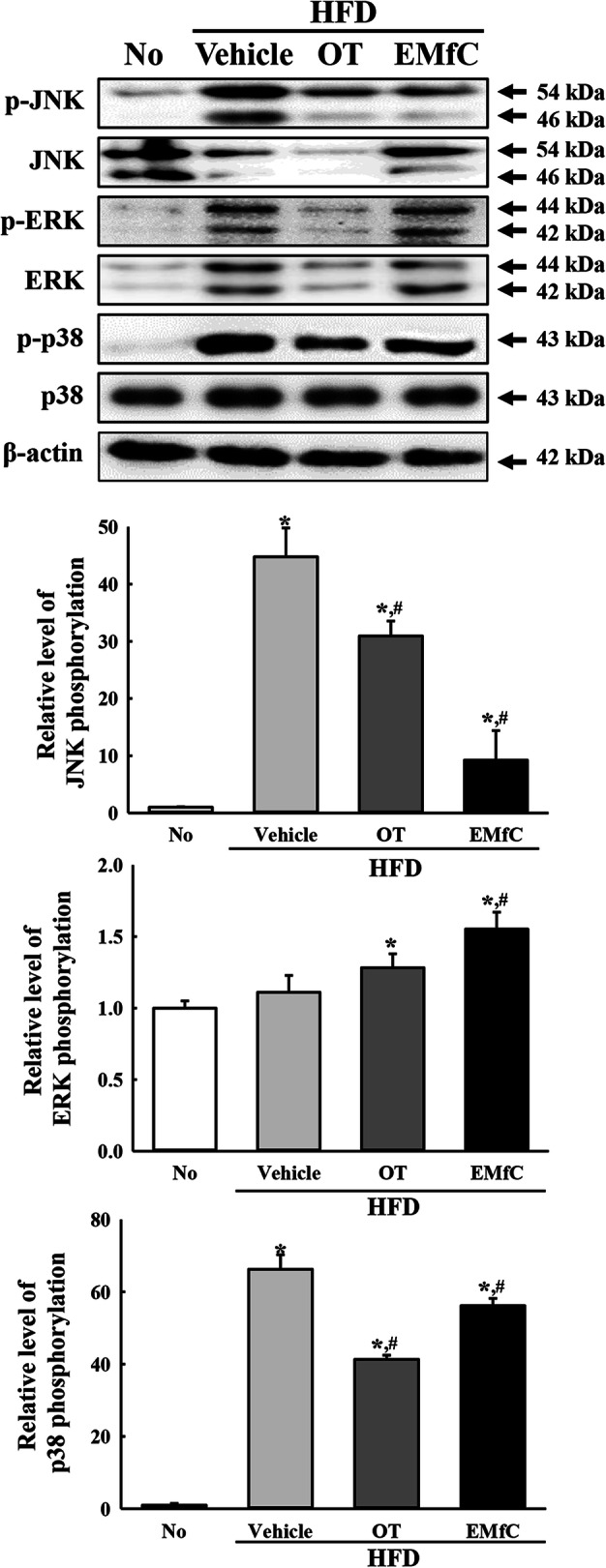
Expression of MAPK signaling pathway in liver tissue. Western blot analysis measured the p-JNK, JNK, p-ERK, ERK, p-p38, p38 and β-actin proteins in total liver homogenate using their specific antibodies. Band intensity of each protein were measured using an imaging densitometer, and the expressions of the six proteins were calculated relative to the intensity of β-actin protein. Three to five mice per group were used in the preparation of liver homogenates, and Western blot analysis was assayed in duplicate for each sample. Data represent the mean ± SD. Asterisk symbol (*) indicates significant *P* < 0.05 compared to the No treated group. Sharp symbol (#) indicates significant *P* < 0.05 compared to the HFD + Vehicle treated group

**Fig. 4:**
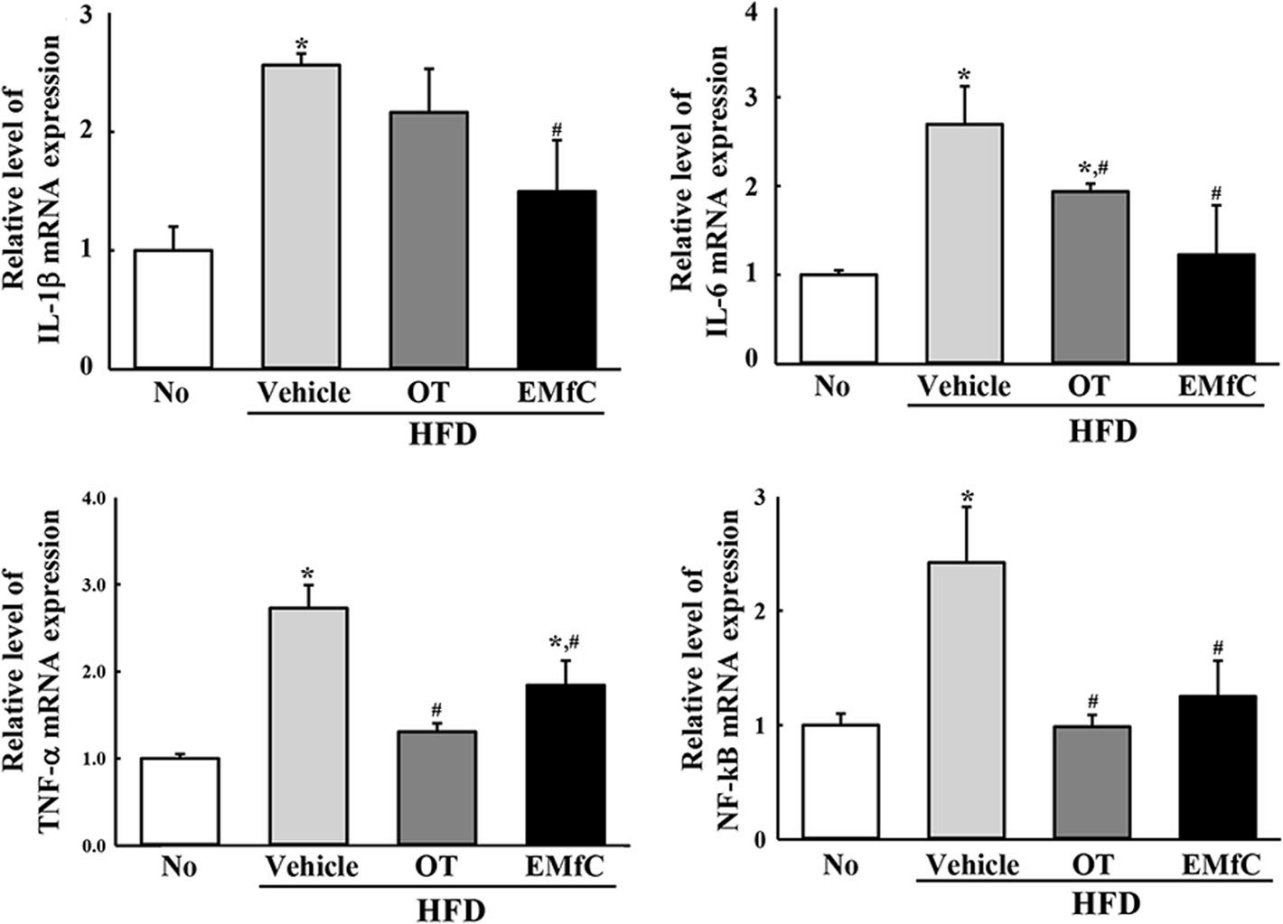
Expression level of pro- and anti-inflammatory cytokines in liver tissue. The levels of IL-1β, IL-6, TNF-α and NF-κB mRNA in the total transcripts of liver tissue were measured by qRT-PCR analyses using their specific primers. The relative levels of the IL-1β, IL-6, TNF-α and NF-κB mRNA were calculated based on the intensity of β-actin as an endogenous control. Three to five mice per group were used in the preparation of the total RNAs, and qRT-PCR analyses was assayed in duplicate for each sample. The values of data represent the mean ± SD. Asterisk symbol (*) indicates significant *P* < 0.05 compared to the No treated group. Sharp symbol (#) indicates significant *P* < 0.05 compared to the HFD + Vehicle treated group

To investigate the effect of EMfC on the regulation of inflammatory response during anti-hepatic steatosis, alterations on mast cells infiltration, expression of inflammatory mediators, inflammatory stimuli-induced MAPK pathway activation, and inflammatory cytokine transcriptions were measured in the liver tissue after administration of EMfC. Remarkably higher numbers of mast cells were stained blue in the HFD + Vehicle treated group than the No treated group. However, the numbers were significantly decreased in the HFD + OT and HFD + EMfC treated groups (72% and 48%, respectively) (Fig. 2a). A similar pattern was observed for mediators in the iNOS-mediated COX-2 induction pathway after EMfC treatment. The levels of iNOS and COX-2 gene transcripts were higher in the HFD + Vehicle treated group than in the No group, but significantly decreased in the HFD + OT and HFD + EMfC treated groups (Fig. 2b). Furthermore, the alteration of iNOS and COX-2 transcript levels were accompanied by a change in MAPK pathway. The phosphorylation levels of ERK, JNK and p38 were variably higher after HFD administration as compared to No treatment. Of the three members, the phosphorylation levels of JNK and p38 were remarkably decreased after EMfC treatment, while the level of ERK was increased (Fig. 3). Moreover, the transcription levels of anti- and pro-inflammatory cytokines were completely reflected as alterations in the MAPK pathway; the transcription levels of IL-1β, IL-6, TNF-α and NF-κB were also very similar in the subset group. The enhanced levels of the four cytokines were decreased in the HFD + OT and HFD + EMfC treated groups, although the rate of decline was varied for each gene (Fig. 4). Taken together, these results indicate that the anti-hepatic steatosis effect of EMfC is associated with the suppression of inflammatory responses in the liver of HFD-induced obesity model.

### Regulation of autophagy and PI3K/AKT/mTOR signaling pathway during anti-inflammatory response of EMfC

**Fig. 5:**
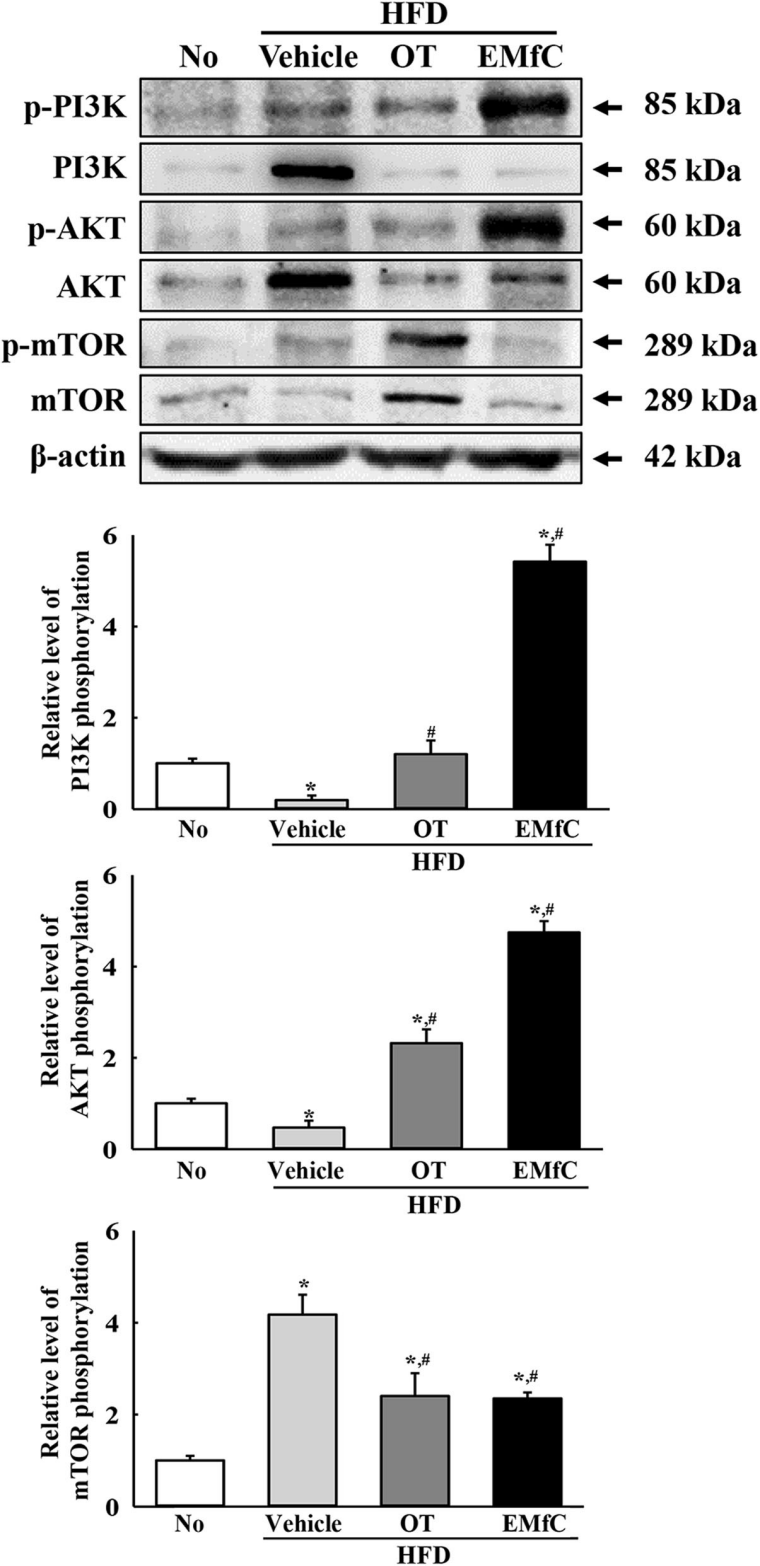
Expression of PI3K/AKT/mTOR signaling pathway in liver tissue. Western blot analysis measured the p-PI3K, PI3K, p-AKT, AKT, p-mTOR, mTOR and β-actin proteins in total liver homogenate using their specific antibodies. Band intensity of each protein were measured using an imaging densitometer, and the expressions of the six proteins were calculated relative to the intensity of β-actin protein. Three to five mice per group were used in the preparation of liver homogenates, and Western blot analysis was assayed in duplicate for each sample. Data represent the mean ± SD. Asterisk symbol (*) indicates significant *P* < 0.05 compared to the No treated group. Sharp symbol (#) indicates significant *P* < 0.05 compared to the HFD + Vehicle treated group

**Fig. 6:**
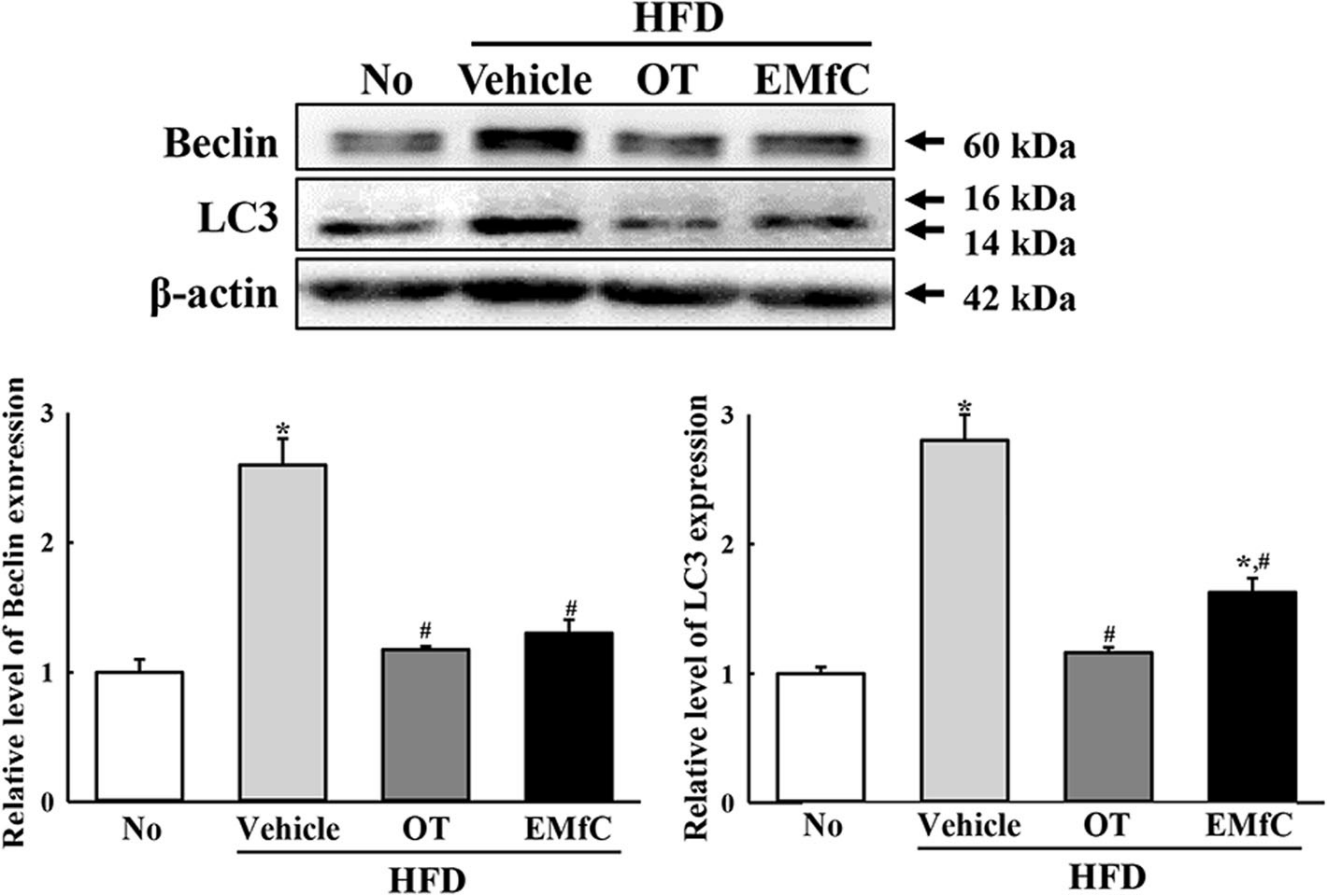
Expression of autophagy regulators in liver tissue. Western blot analysis measured the Beclin, LC3-I/II and β-actin proteins in total liver homogenate using their specific antibodies. Band intensity of each protein were measured using an imaging densitometer, and the expressions of the two proteins were calculated relative to the intensity of β-actin protein. Three to five mice per group were used in the preparation of liver homogenates, and Western blot analysis was assayed in duplicate for each sample. Data represent the mean ± SD. Asterisk symbol (*) indicates significant *P* < 0.05 compared to the No treated group. Sharp symbol (#) indicates significant *P* < 0.05 compared to the HFD + Vehicle treated group

**Fig. 7:**
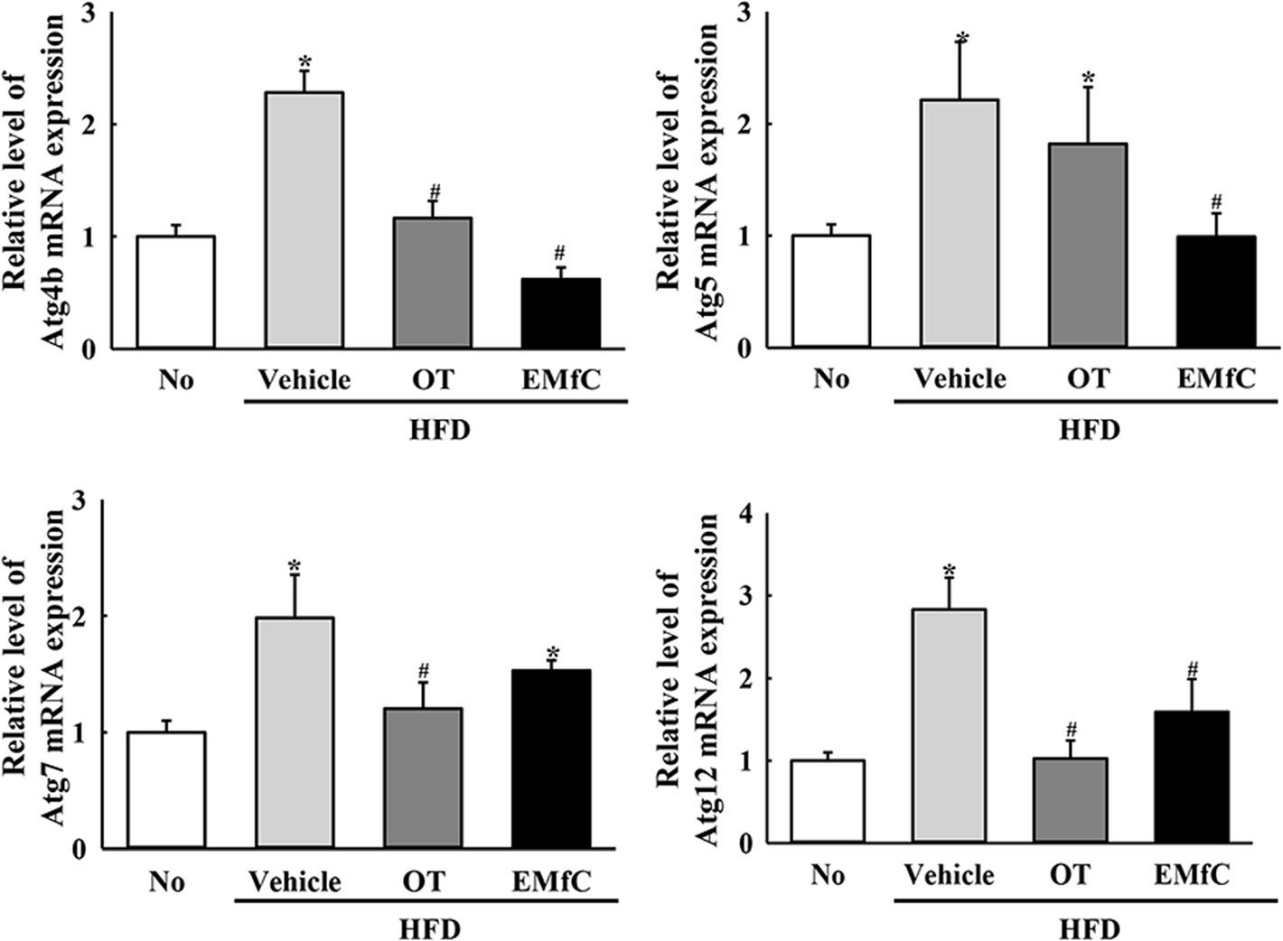
Transcription of autophagy related genes in liver tissue. The levels of Atg4b, Atg5, Atg7, and Atg12 mRNA in the total transcripts of liver tissue were measured by qRT-PCR analyses using their specific primers. The relative levels of the Atg4b, Atg5, Atg7, and Atg12 mRNA were calculated based on the intensity of β-actin as an endogenous control. Three to five mice per group were used in the preparation of the total RNAs, and qRT-PCR analyses was assayed in duplicate for each sample. The values of data represent the mean ± SD. Asterisk symbol (*) indicates significant *P* < 0.05 compared to the No treated group. Sharp symbol (#) indicates significant *P* < 0.05 compared to the HFD + Vehicle treated group

We next investigated the role of autophagy in the sequential inhibition of inflammatory response by applying EMfC to hepatic steatosis of HFD-induced obesity model. Significant alterations were observed in the PI3K/AKT/mTOR signaling pathway after treatment with EMfC. The decrease in the phosphorylation levels of PI3K and AKT in the HFD + Vehicle treated group were observed to increase in the HFD + EMfC treated group, whereas the reverse was true for the phosphorylation level of mTOR (Fig. 5). Furthermore, we investigated whether the alteration of PI3K/AKT/mTOR signaling pathway after EMfC exposure is accompanied with an activation of autophagy. To achieve this, the expression levels of genes and proteins included in autophagic flux were examined in the liver of HFD + EMfC treated mice because these proteins were degraded by autophagy. The proteins levels of Beclin and LC3 were higher in the HFD + Vehicle treated mice than the No treated group. However, levels were significantly decreased in the HFD + OT and HFD + EMfC treated groups (Fig. 6). In addtion, a similar response was observed in the mRNA expression levels of four autophagy related genes including Atg4b, Atg5, Atg7, and Atg12. Under conditions of obesity, levels of all genes were increased in the HFD + Vehicle group as compared to the No treated group. However, the transcript levels of these genes were remarkably recovered in the HFD + OT and HFD + EMfC groups, although at varied degrees (Fig. 7). These results indicate that activation of the autophagy machinery in hepatic steatosis of HFD-induced obesity model is associated with the anti-inflammatory response of EMfC, including the AKT/mTOR signaling pathway and regulator proteins.

## Discussion

The beneficial effects of mulberry leaves have been investigated for the regulation of lipid metabolism, although there are few studies for inflammation and autophagy. Previous studies report that the extract of these leaves downregulate the lipogenesis-related genes in rats after 7 weeks treatment, and remarkably reduces the expression of the lipoprotein-related protein and accumulation of lipid in 3T3-L1 adipocytes [34]. Also, the extract of mulberry leaves induces decrease in the adipocyte differentitation in white adipose tissue of db/db mice through suppression of oxidative stress [41, 42]. Fermented leaves were reported to prevent fat accumulation in the HFD-induced obese C57BL/6 mice through stimulation of lipolysis and by inhibition of lipogenesis [32]. In this study, we investigated the molecular mechanism of inflammation and autophagy, based on the inhibitory effect exerted by EMfC for fat accumulation. We believe that the current study results provide first evidence of the molecular mechanism for anti-hepatic steatosis, where EMfC may be associated with the regulation of inflammation and autophagy in the HDF-induced obesity model.

In a variety of studies to evaluate anti-obesity effect of natural products, several chemical drugs have been used as positive control for obesity management. Among these, OT has been well known an inhibitor of the gastrointestinal lipase that decrease with 30% of fat accumulation, body weight and improve cardiovascular risk factors of obese patients [43–45]. Also, OT treatment is associated with significant improvement in blood pressure, TC, LDL, the incidence of type II diabetes and the insulin sensitivity [46–48]. In HFD-induced obesity model, OT was significantly decreased the level of adipogenesis regulators including PPAR, C/EBP, FAS and aP2, while it increased the phosphorylation of perilipin and expression of ATGL [32]. Although OT has above therapeutic effects, it cannot induce any serious systemic side effects during long periods of intake [49]. Because of these properties on toxicity and therapeutic mechanism, OT is most commonly used as a positive control in many studies identifying novel natural products with anti-obesity activity. Therefore, our study had selected OT as standard drug for evaluation of EMfC. On the other hand, metformin has been considered as one of another control drugs because it decreases hyperinsulinemia, insulin resistance and adiposity in obesity and type II diabetes [50–52].

Among all the inflammatory cytokines, IL-6 is considered to a useful marker for obesity-induced inflammation and is secreted from Kupffer cells and HSC of the liver tissue [53, 54]. IL-6 and TNF-α enhance the hepatic production of C-creative protein (CRP), which is increased under conditions of obesity [55]. Furthermore, other cytokines such as IL-1β, OSM (oncostatin M) and LIF (leukaemia inhibitory factor) are induced with the production of CRP (C-reactive protein) [56]. A significant decrease of IL-6 and TNF-α levels were observed in the liver of HFD-induced obesity model after exposure to several natural products [11–13] and their fermented products [14]. In our study, treatment with EMfC prepared by fermentation, suppresses the IL-6 and TNF-α levels in the liver of HFD-induced C57BL/6J mice. The results of the present study were agree with earlier studies that report suppression of the expression of key inflammatory cytokines in the HFD-induced model by natural products, although the suppression rates were varied.

Previous pharmacological studies have demonstrated the potential of herbal medicine extracts and natural products in regulating the autophagy flux during inhibition of hepatic steatosis, since the activation of autophagy is considered a promising strategy to inhibit lipid accumulation in the liver [57]. Ginsenoside Rb2 partly activates the autophagic pathway through inhibition of the AMPK or SIRT1 pathway in db/db mice [58]. Also, resveratrol and akebia saponin D prevent hepatic steatosis, accompanied with up-regulation of autophagy markers such as LC3-II, Beclin 1 and p62 [59, 60]. Similar effects on the induction of autophagy and prevention of hepatic steatosis have been reported in obesity animals and hepatic cell lines after exposure to the bergamot polyphenol fraction (BPF) and capsaicin [29, 60, 61]. It is also documented that induction of hepatic autophagy enhances the effects of quercetin, metformin and *Eucommia ulmoides* leaf extract [30, 62, 63]. In this study, hepatic autophagy is significantly activated with EMfC in the HFD-induced obesity model during anti-hepatic steatosis activity. The expression of autophagy marker proteins and PI3K/AKT/mTOR signaling pathway remarkably recover after exposure to EMfC. These results are consistent with previous studies, although further research on the mechanism of action is required. The results of the present study especially provide novel evidence that the anti-hepatic steatosis of EMfC may be associated with the amelioration of the induction step and late suppression step on the autophagic flux and autophagosomes formation, although HFD treatment also prevents these processes [57].

Among the several autophagy marker proteins, LC3-II is widely used as one of the autophagosome markers, since the amount of LC3-II reflects the number of autophagosomes and autophagy-related structures [57, 64]. LC3 is localized at the membranes and autophagosomes, and processed into LC3-I and LC3-II through the C-terminal cleavage of Atg4 and conjugation of phosphatidylethanolamine [65]. Especially, enhanced levels of LC3-II represent not only autophagy induction, but also a blockade in the autophagosome maturation [66]. Various animal models including ob/ob mice [60], the HFD-treated C57BL/6J mice [67] and HFD-treated Wistar rat [68] show increased levels of LC3-II during hepatic steatosis. A similar increase in the level of LC3-II was observed in our current study using the HFD-induced obesity model, although the rate of enhancement was varied. These results indicate that LC3-II is a key marker for anti-hepatic steatosis induced by fermented natural products, including EMfC.

In this study, we investigated the role of EMfC on activation mechanism of autophagy during anti-hepatic steatosis. EMfC induced activation of autophagy via the PI3K/AKT/mTOR signaling pathway in the HFD-induced obesity model as shown Figs. 5 and 6. A similar regulatory mechanism was observed after the treatment of several compounds with anti-obesity activity. Akebia saponin D (ASD) treatment prevent the oleic acid (OA)-induced upregulation of LC3-II, Beclin and P62 expression as well as accumulation of lipid droplets in ob/ob mice [59]. Also, *Lycium barbarum* polysaccharides induced the down-regulation of p-mTOR and p62 level, while the expression level of Beclin and LC3 were enhanced in HFD-induced NASH rat model [69]. The decrease of p-mTOR and p-AKT level was detected in dimethylnitrosamine (DMN)-induced hepatic fibrosis model after treatment of dioscin [70]. However, some differences were reported on the regulation of autophagy markers, key regulators of autophagy and signaling pathway in animal model treated with anti-obesity products although autophagy flux was activated. Several key regulators of autophagy including Beclin 1, ATG7, LAMP1 and LAMP2 were upregulated in HFD-fed C57BL/6J mice after astaxanthin treatment, while the phosphorylation level of Akt decreased in same group [71]. Ginsenoside Rb2 induce the enhancement of LC3-II and decline of mTOR phosphorylation in db/db mice [57]. Resveratrol prevent hepatic steatosis and increase the expression of LC3 proteins in HFD-fed Wistar rats [58]. Also, the increased level of LC3 and Beclin 1 protein was detected in cafeteria (CAF) diet-fed WIST rats treated with bergamot polyphenol fraction (BPF) during the reduce of hepatic steatosis and total lipid content [29]. Furthermore, EGCG treatment induce a similar effect on the regulation of autophagy such as activation of autophagy and upregulation of autophagic biomarkers [72]. Therefore, we thought that these differences can be attributed to variety in the composition of treated products, pathological severity of hepatic steatosis and treatment conditions between each study.

It is difficult to clearly verify the bioactive compound candidate responsible for the improvement effects of EMfC on the inflammatory response and autophagy regulation. However, several previous studies provided a clue for correlation between bioactive compounds and these effects. The first candidate is cordycepin because it show anti-obesity effects as well as multiple-biological effects including modulation of immune response, inhibition of tumor growth, hypotensive and vasorelaxation activities, and promoting secretion of adrenal hormone [73]. This compound was detected in the HPLC chromatogram of EMfC under the optimal conditions [33]. Also, other candidates are 4 compounds including mulberroside A, 5,7,2′-trihydroxyflavanone-4′-O-β-d-glucoside, albanols A and albanols B in 70% alcohol extract fractions of the Egyptian *M. alba*. They inhibited LDL atherogenic modifications and lipid peroxides formation in hypercholesterolemic rats [74]. In this study, we did not investigate what the bioactive compound in EMfC induce the anti-hepatic steatosis through the regulation of the inflammatory response and autophagy pathway. However, the previous study of our team provided few evidences that cordycepin has been considered as key compound for these effects although further research will be needed.

Generally, oxidative balance impairment, NAFLD-related fat accumulation and mitochondrial dysfunction have been considered as one of major target for preventing the progression of hepatic steatosis in various studies [75]. During fat accumulation in hepatocytes, ROS production and lipid peroxidation were excessively enhanced, and subsequently lead to the formation of other reactive metabolites including 4-HNE and MDA [76, 77]. Therefore, several fermented products with high antioxidant activity such as fermented green tea extracts and garlic extract improved and prevent hepatic steatosis of HFD-induced model [78, 79]. However, in this study, we did not investigate about oxidative stress and free radical scavenging activity of EMfC in liver tissue of HFD-induced obesity model because this extract showed a relatively low level of DPPH scavenging activity (IC_50_ = 579 μg/mL) in preliminary study (Data not shown). Instead, we focused on the improvement effect and action mechanism of EMfC in the inflammation and autophagy although further analysis for detail mechanism such as inflammasome is required.

Meanwhile, various animal models such as ob/ob mice and Zucker (fa/fa) rats have been used to study the pathogenesis and therapeutic drugs for obesity and diabetes although they did not completely reflect human diseases [80]. These model for obesity can classified into mutation type being based on mutation or manipulation of individual genes and genetically intact type exposing obesogenic environment such as HFD [81]. Among these, HFD-induced obesity model was firstly reported as obesogenic environment-induced model in 1959 [82]. They showed the reduction of insulin and leptin sensitivity, alteration of neuropeptide expression, hyperglycemia and compromised β-cell function [83–85]. Especially, HFD treatment for 12 weeks induce some significant alterations on the pathology and biochemical markers for hepatic steatosis. In the liver tissue of above mice, adipocyte size, percentage of crown-like structures, severity of hepatic steatosis, and number of inflammatory foci were remarkably increased compared with normal diet (ND)-fed mice. A similar enhancement was detected on the levels of serum biochemical markers including TC, glucose, and aminotransferases (AST and ALT) in HFD treated mice for 12 weeks [32, 86–88]. Also, the increase of insulin and leptin concentration were detected in the same mice [89]. Base on above reports, 12-week period had been selected as the optimum period for inducing hepatic steatosis of C57BL/6N mice in this study. However, HFD-induced model provides limited information because only one high fat formula employed to induce obesity. The treatment of diet based on saturated fatty acids exhibit the typical phenotypes of HFD-induced obesity, while the treatment of diet containing polyunsaturated ω-3 fatty acid show some beneficial effects on the body position and insulin action [90]. Therefore, more studies are necessary to clarify the influence of the specific fat composition in HFD-induced obesity model.

## Conclusions

**Fig. 8:**
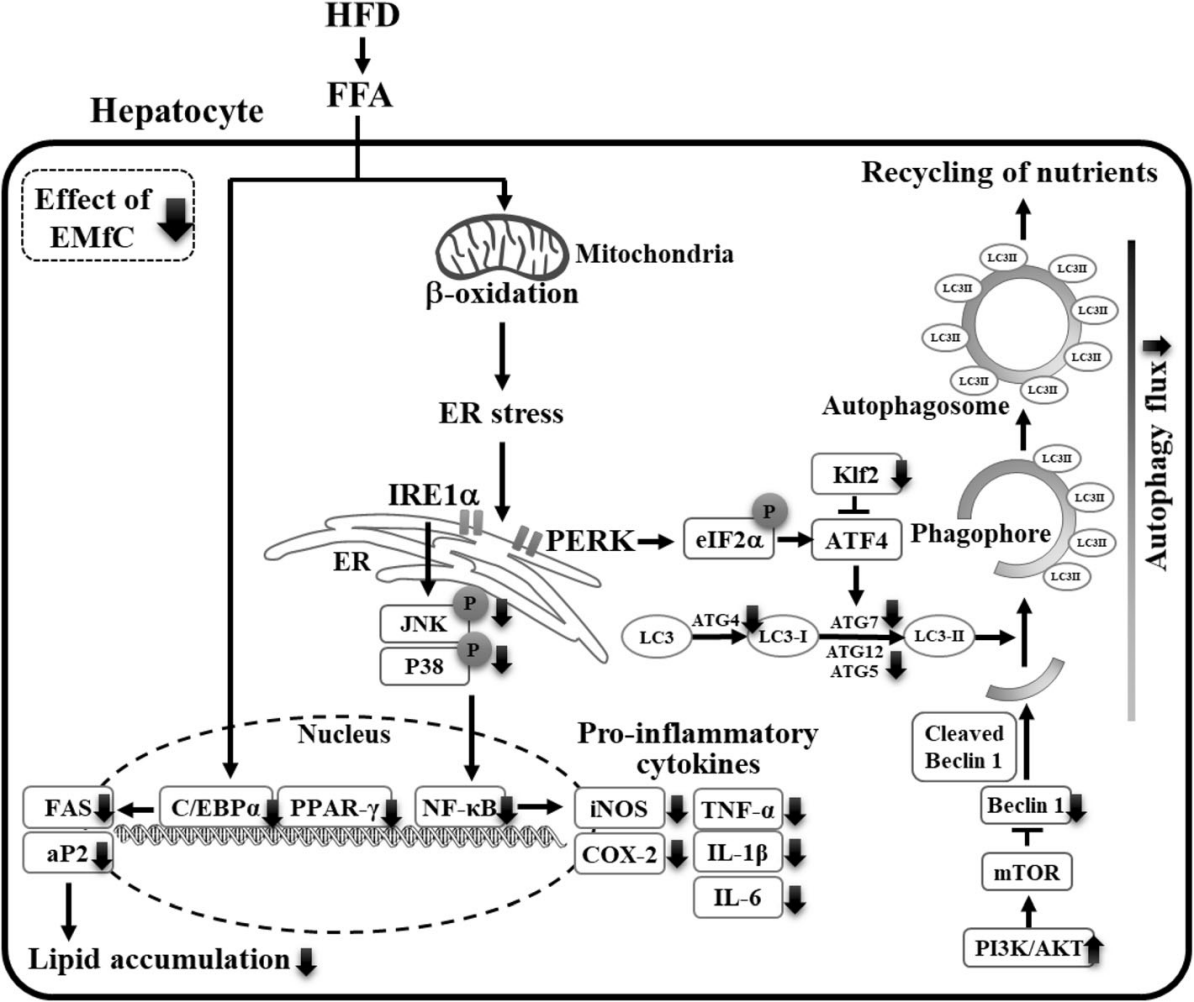
Proposed mechanism of EMfC action in amelioration of inflammatory response and autophagy pathway during anti-hepatic steatosis in HFD-induced obesity mice. In this scheme, the anti-hepatic steatosis effects of EMfC are thought to be exerted the suppression of inflammatory response and autophagy flux in hepatocytes during HFD-induced β-oxidation

In conclusion, the current study demonstrates that EMfC exerts anti-hepatic steatosis activity by successfully reducing the inflammatory response, including mast cell infiltration, inflammatory mediators and cytokines, in the liver tissue of HFD-induced obese mice. In addition, the anti-inflammatory response of EMfC is mediated by autophagy flux, PI3K/AKT/mTOR signaling pathway and autophagic gene transcripts. The regulatory effects of EMfC on the inflammatory response and autophagy during the suppression of hepatic steatosis indicates the potential of EMfC as an anti-obesity drug that results in lipolysis (Fig. 8).

## Supplementary information

**Additional file 1.**

**Additional file 2.**

## Data Availability

The extract of mulberry leaves fermented with *Cordyceps militaris* (EMfC) used in this study are available from the corresponding author on reasonable request.

## Abbreviations

AKT: Protein kinase B
Atg: Autophagy-related gene
COX: Cyclooxygenase
EMfC: Extract of mulberry leaves fermented with *Cordyceps militaris*
ERK: Extracellular signal-regulated kinase
HFD: High fat diet
IL: Interleukin
iNOS: Inducible nitric oxide synthase
JNK: c-Jun N-terminal kinases
Klf2: Krüppel-like Factor 2
LC3: Light chain 3
MAPK: Mitogen-activated protein kinase
mTOR: Mammalian target of rapamycin
NF-κB: Nuclear Factor-kappaB
OT: Orlistat
PI3K: Phosphoinositide 3-kinase
TNF-α: Tumor necrosis factor-α
