# Supplementary material for: Fermented mulberry (Morus alba) leaves suppress high fat diet-induced hepatic steatosis through amelioration of the inflammatory response and autophagy pathway
Source: BMC Complement Med Ther. 2020 Sep 18;20:283. doi: 10.1186/s12906-020-03076-2 (PMC7501671; doi:10.1186/s12906-020-03076-2)
Supplement: Supplementary file 1 — Additional file 1. [file 12906_2020_3076_MOESM1_ESM.pdf]

Quality of Extracted RNA

group		concentration (ng/ul)	260/280	A260	A280
No	1	849.0	1.81	21.23	11.7
	2	864.9	1.81	21.62	11.91
	3	808.3	1.79	20.21	11.27
	3	711.1	1.78	17.78	10
Vehicle	1	854.8	1.82	21.37	11.76
	2	773.3	1.8	19.33	10.73
	3	819.8	1.81	20.47	11.31
	4	870.2	1.85	20.794	11.24
OT	1	856.2	1.78	19.3842	10.89
	2	814.9	1.84	21.3072	11.58
	3	863.3	1.86	21.3714	11.49
	4	845.7	1.83	20.7705	11.35
EMfC	1	862.5	1.84	20.056	10.9
	2	845.4	1.81	21.0684	11.64
	3	867.3	1.85	20.7385	11.21
	4	844.3	1.82	20.111	11.05

ACTIN

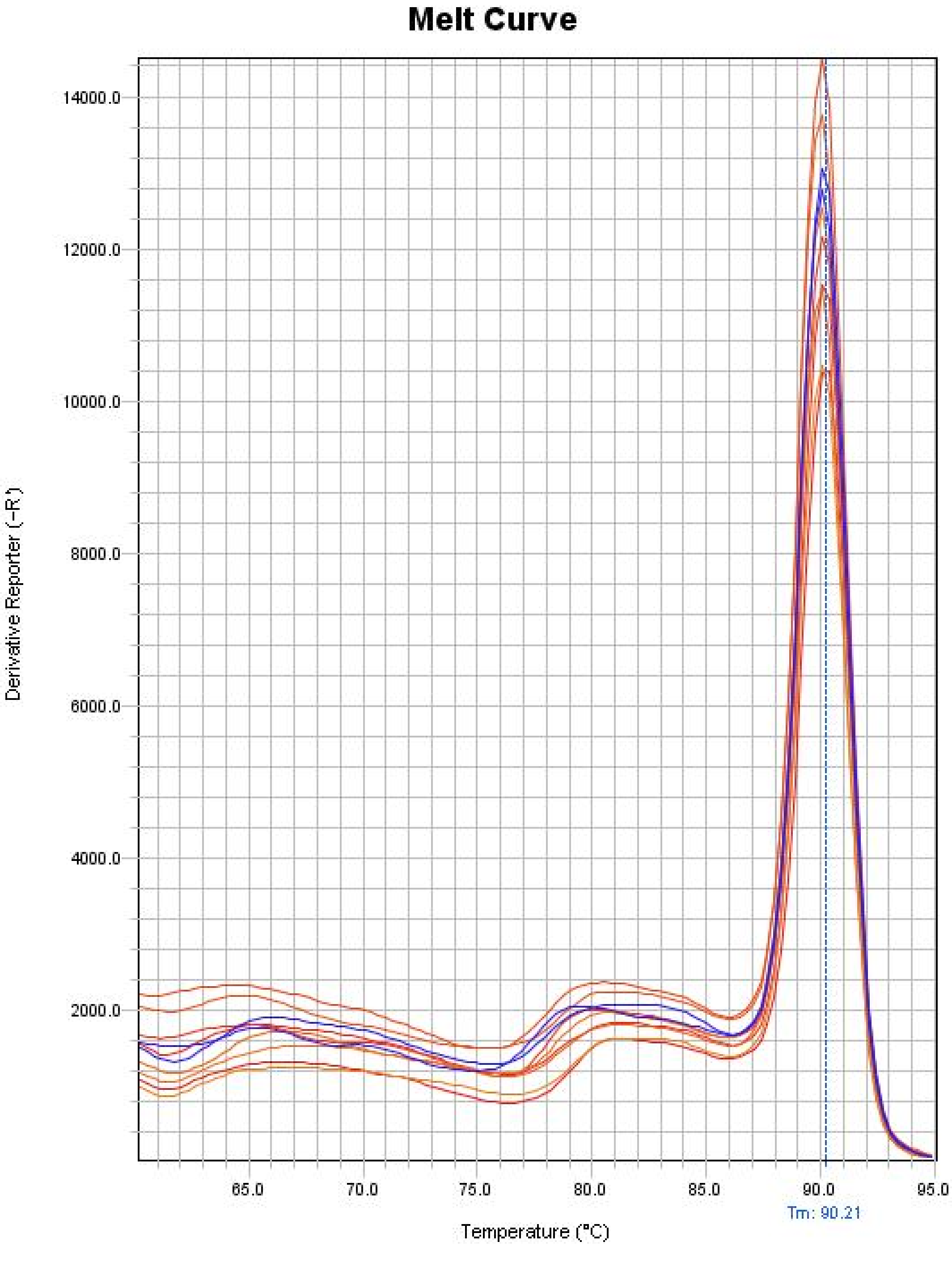

Melt Curve
14000.0
12000.0
10000.0
8000.0
6000.0
4000.0
2000.0
Derivative Reporter (−R')
65.0
70.0
75.0
80.0
85.0
90.0
95.0
Tm: 90.21
Temperature (°C)

Fig1_ap2

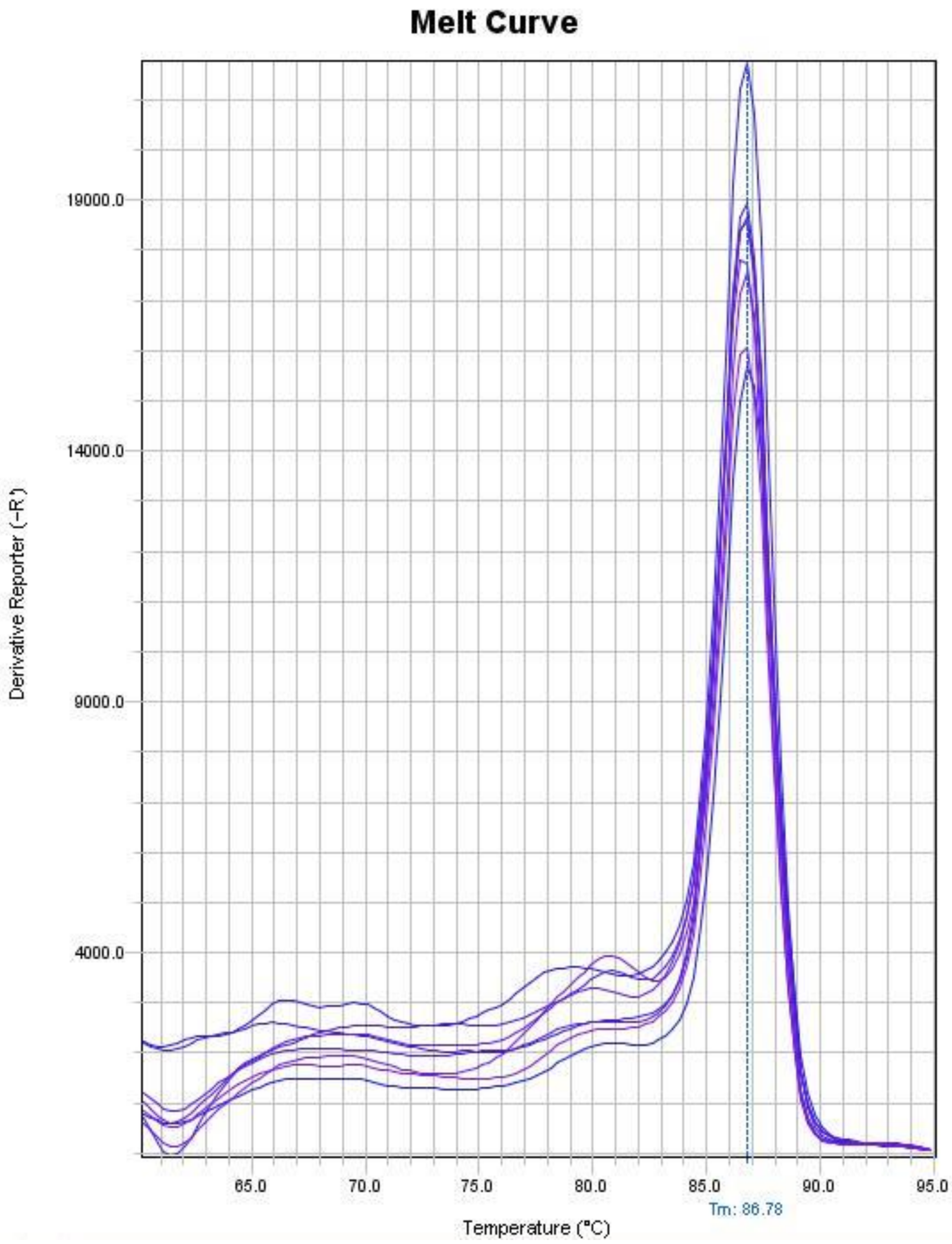
Melt Curve
19000.0
14000.0
9000.0
4000.0
Derivative Reporter (−R')
65.0
70.0
75.0
80.0
85.0
90.0
95.0
Tm: 86.78
Temperature (°C)

Fig1_CEBPa

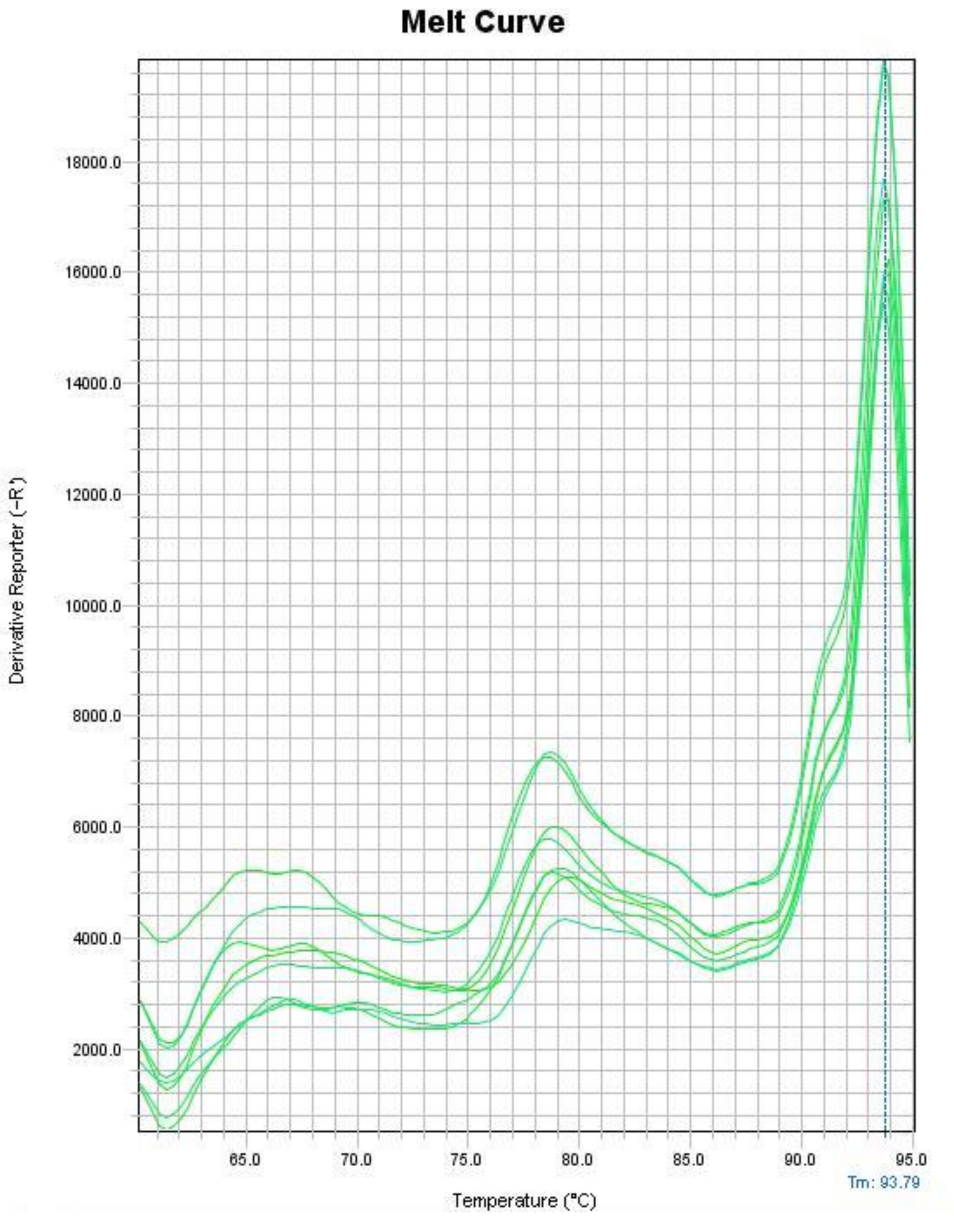

Melt Curve
18000.0
16000.0
14000.0
12000.0
10000.0
8000.0
6000.0
4000.0
2000.0
Derivative Reporter (-R')
65.0
70.0
75.0
80.0
85.0
90.0
95.0
Tm: 93.79
Temperature (°C)

Fig1_klf2

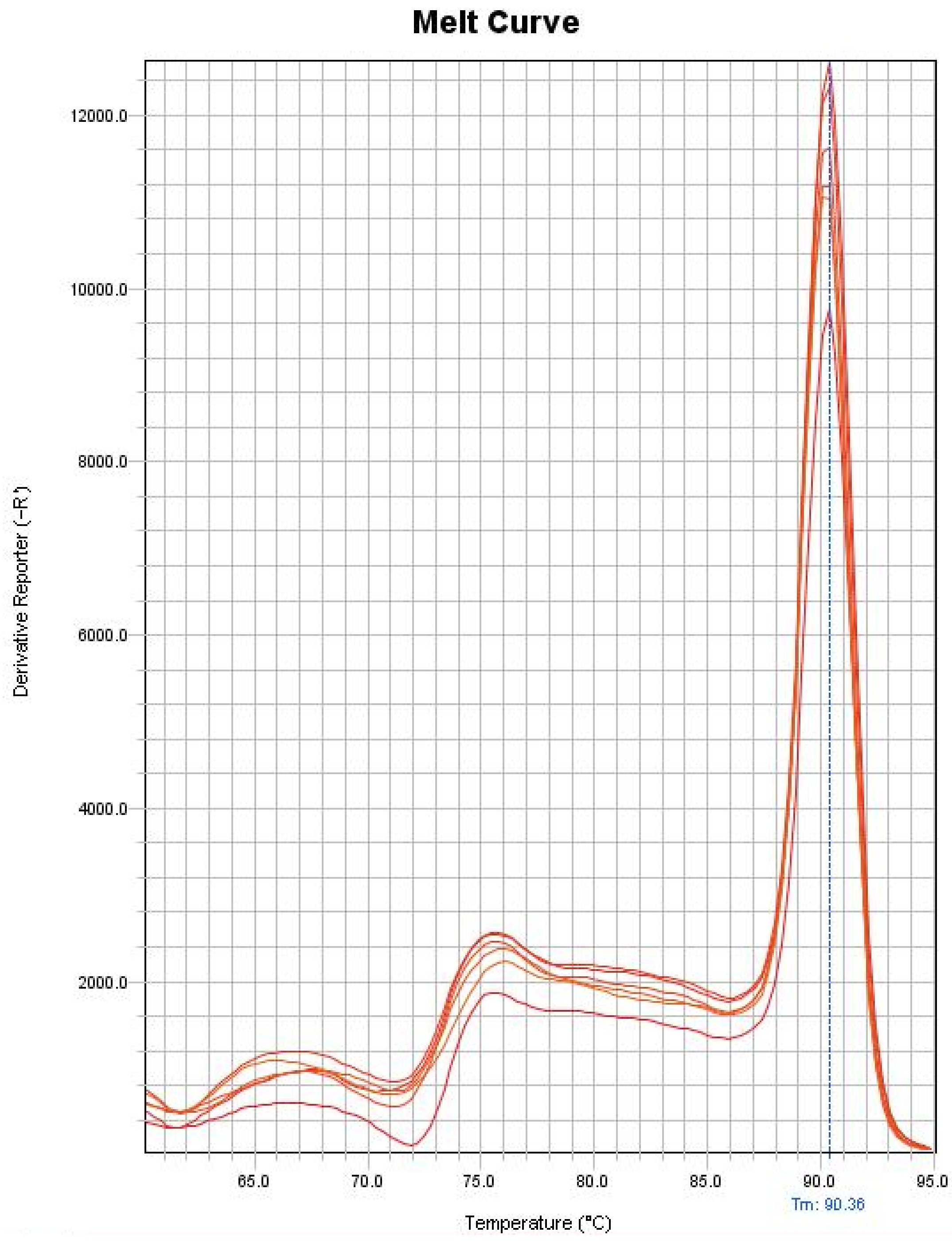
Melt Curve
12000.0
10000.0
8000.0
6000.0
4000.0
2000.0
Derivative Reporter (−R')
65.0
70.0
75.0
80.0
85.0
90.0
95.0
Tm: 90.36
Temperature (°C)

Fig1_PPAR-r

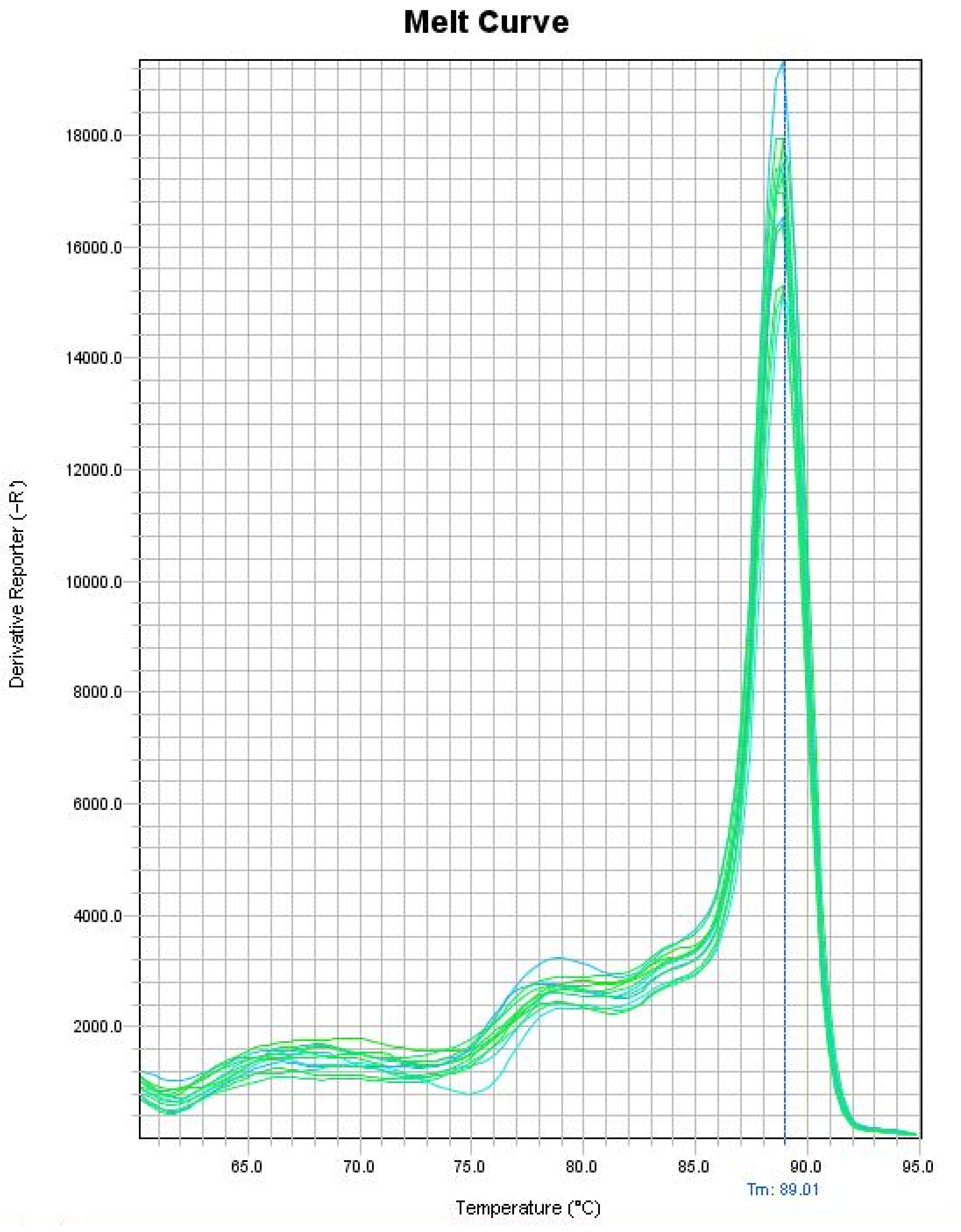

Melt Curve
18000.0
16000.0
14000.0
12000.0
10000.0
8000.0
6000.0
4000.0
2000.0
Derivative Reporter (-R')
65.0
70.0
75.0
80.0
85.0
90.0
95.0
Tm: 89.01
Temperature (°C)

Fig2_COX2

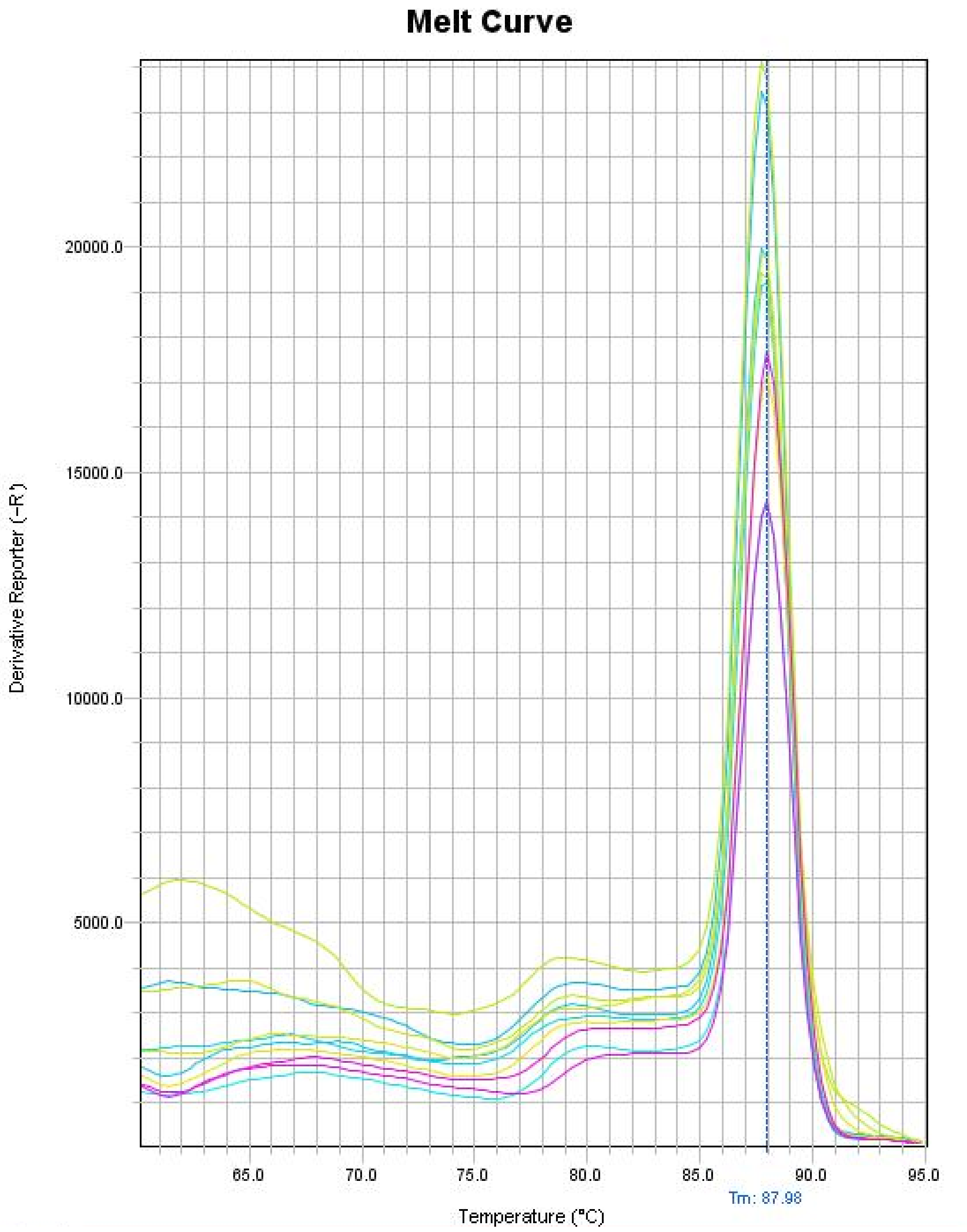
Melt Curve
Derivative Reporter (−R')
20000.0
15000.0
10000.0
5000.0
65.0
70.0
75.0
80.0
85.0
90.0
95.0
Tm: 87.98
Temperature (°C)

Fig2_INOS

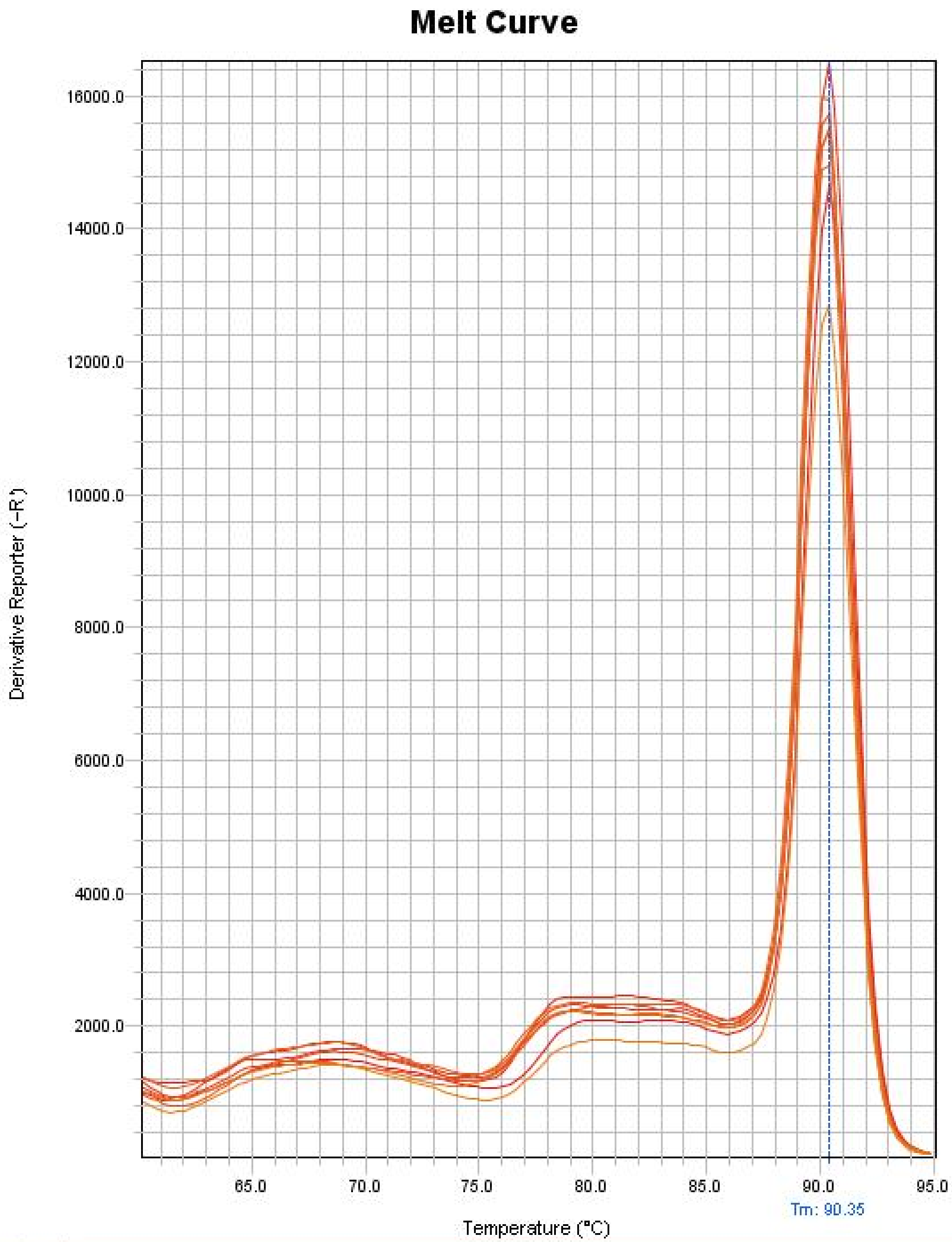

Melt Curve
16000.0
14000.0
12000.0
10000.0
8000.0
6000.0
4000.0
2000.0
Derivative Reporter (-R')
65.0
70.0
75.0
80.0
85.0
90.0
95.0
Tm: 90.35
Temperature (°C)

Fig4_IL-1B

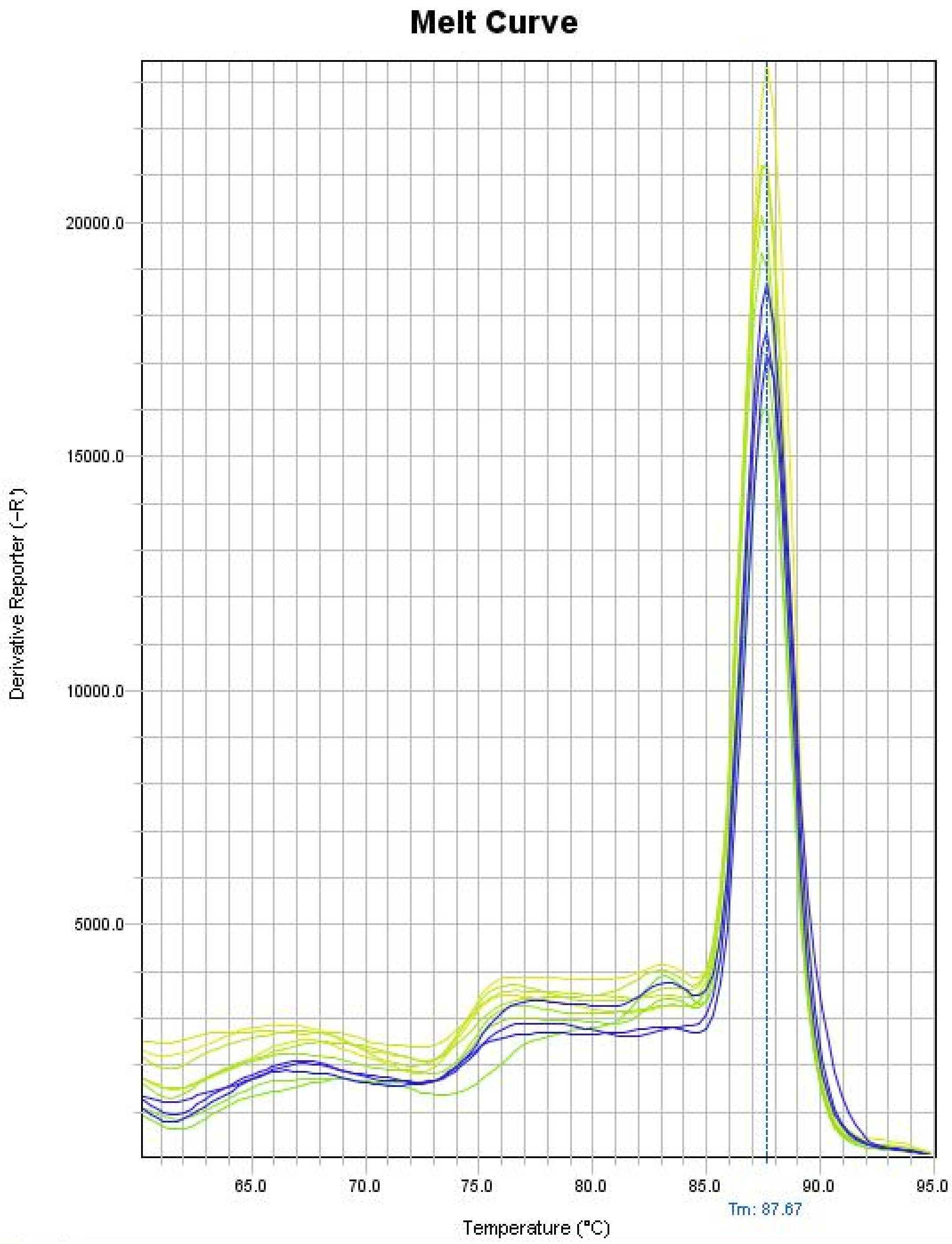

Melt Curve
20000.0
15000.0
10000.0
5000.0
Derivative Reporter (-R')
65.0
70.0
75.0
80.0
85.0
90.0
95.0
Tm: 87.67
Temperature (°C)

Fig4_IL6

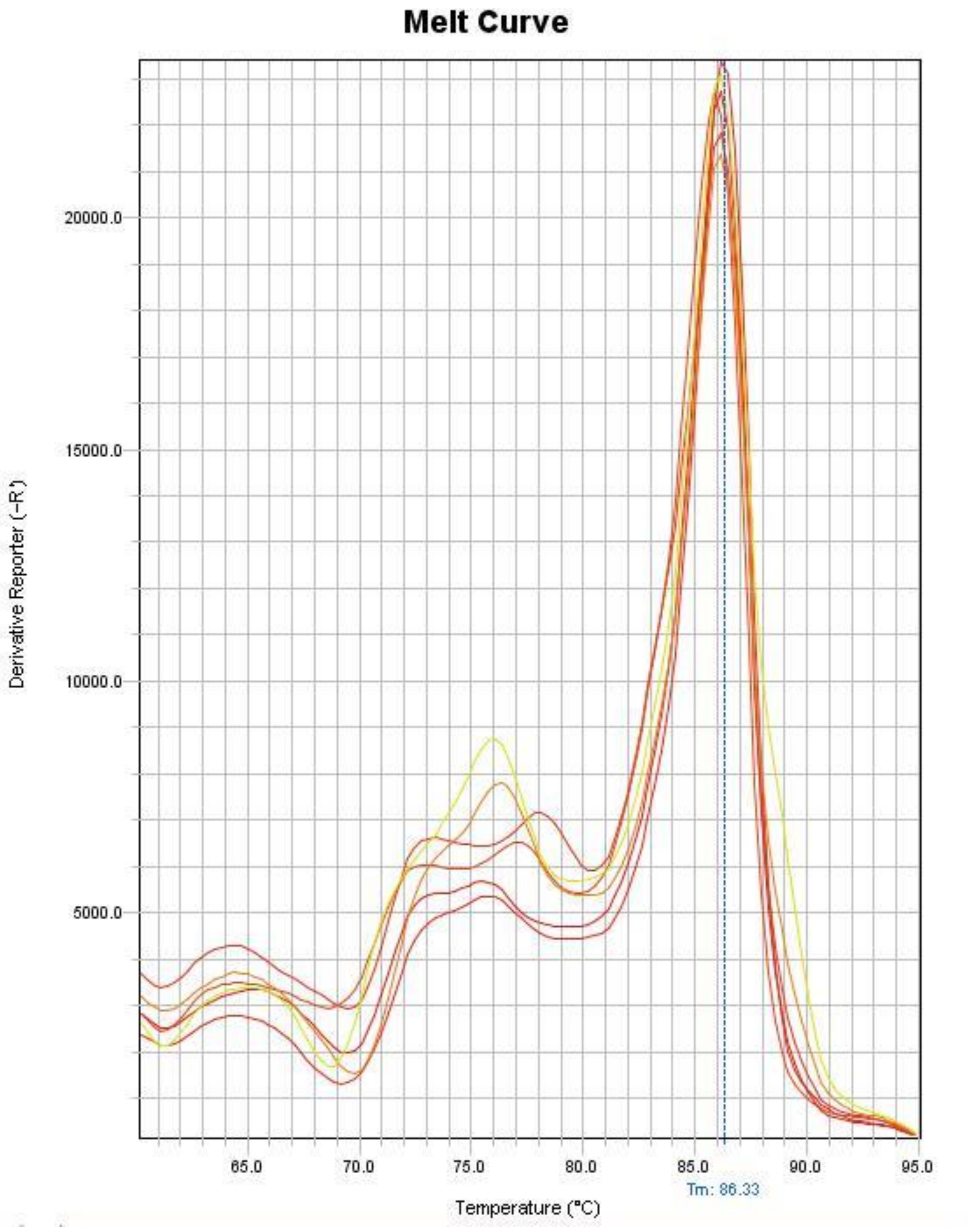
Melt Curve
Derivative Reporter (-R')
20000.0
15000.0
10000.0
5000.0
65.0
70.0
75.0
80.0
85.0
90.0
95.0
Tm: 86.33
Temperature (°C)

Fig4_NFKB

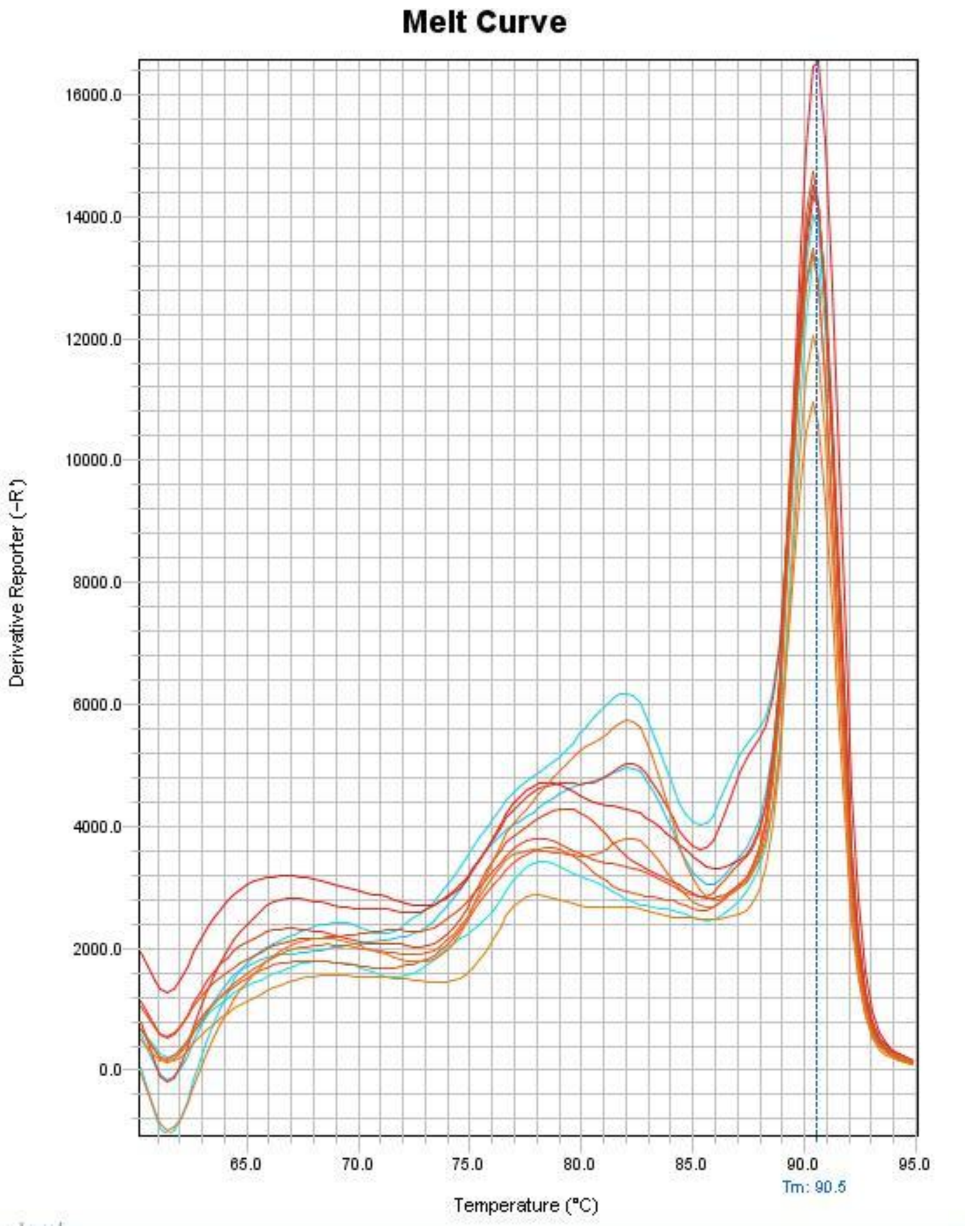
Melt Curve
Derivative Reporter (−R')
16000.0
14000.0
12000.0
10000.0
8000.0
6000.0
4000.0
2000.0
0.0
65.0
70.0
75.0
80.0
85.0
90.0
95.0
Tm: 90.5
Temperature (°C)

Fig4_TNFA

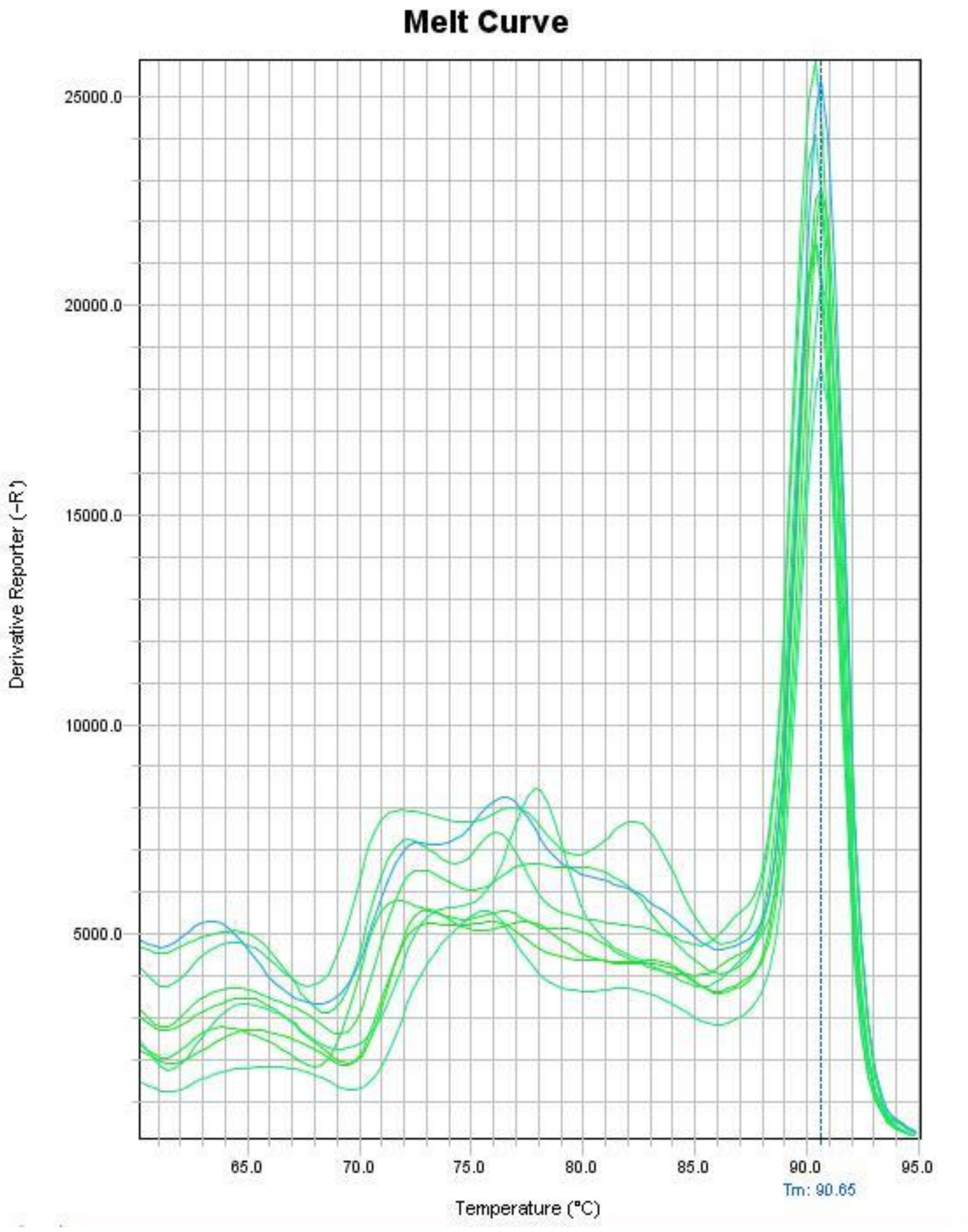
Melt Curve
Derivative Reporter (-R')
25000.0
20000.0
15000.0
10000.0
5000.0
65.0
70.0
75.0
80.0
85.0
90.0
95.0
Tm: 90.65
Temperature (°C)

Fig5_ATG4b

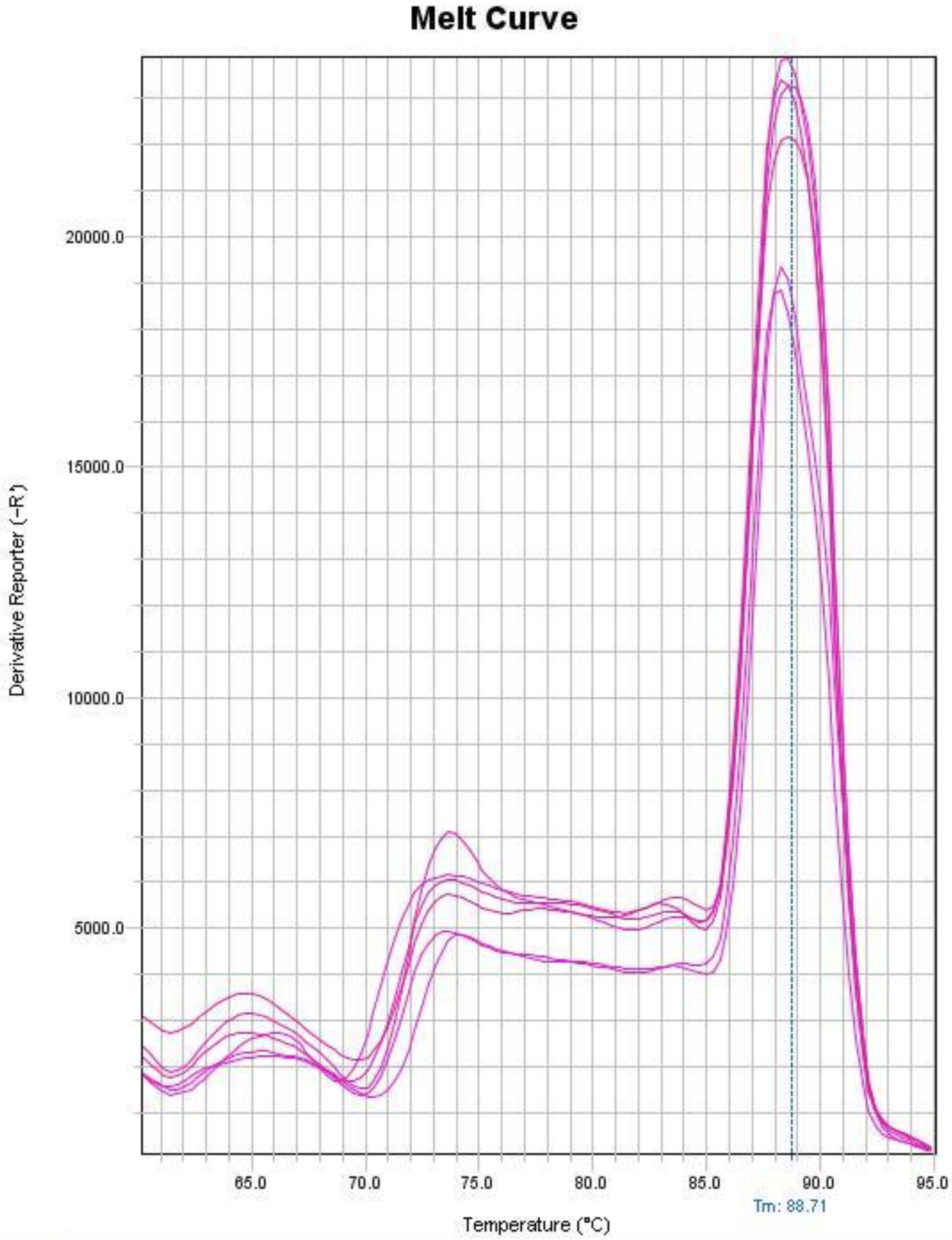
Melt Curve
Derivative Reporter (-R')
20000.0
15000.0
10000.0
5000.0
65.0
70.0
75.0
80.0
85.0
90.0
95.0
Tm: 88.71
Temperature (°C)

Fig5_ATG5

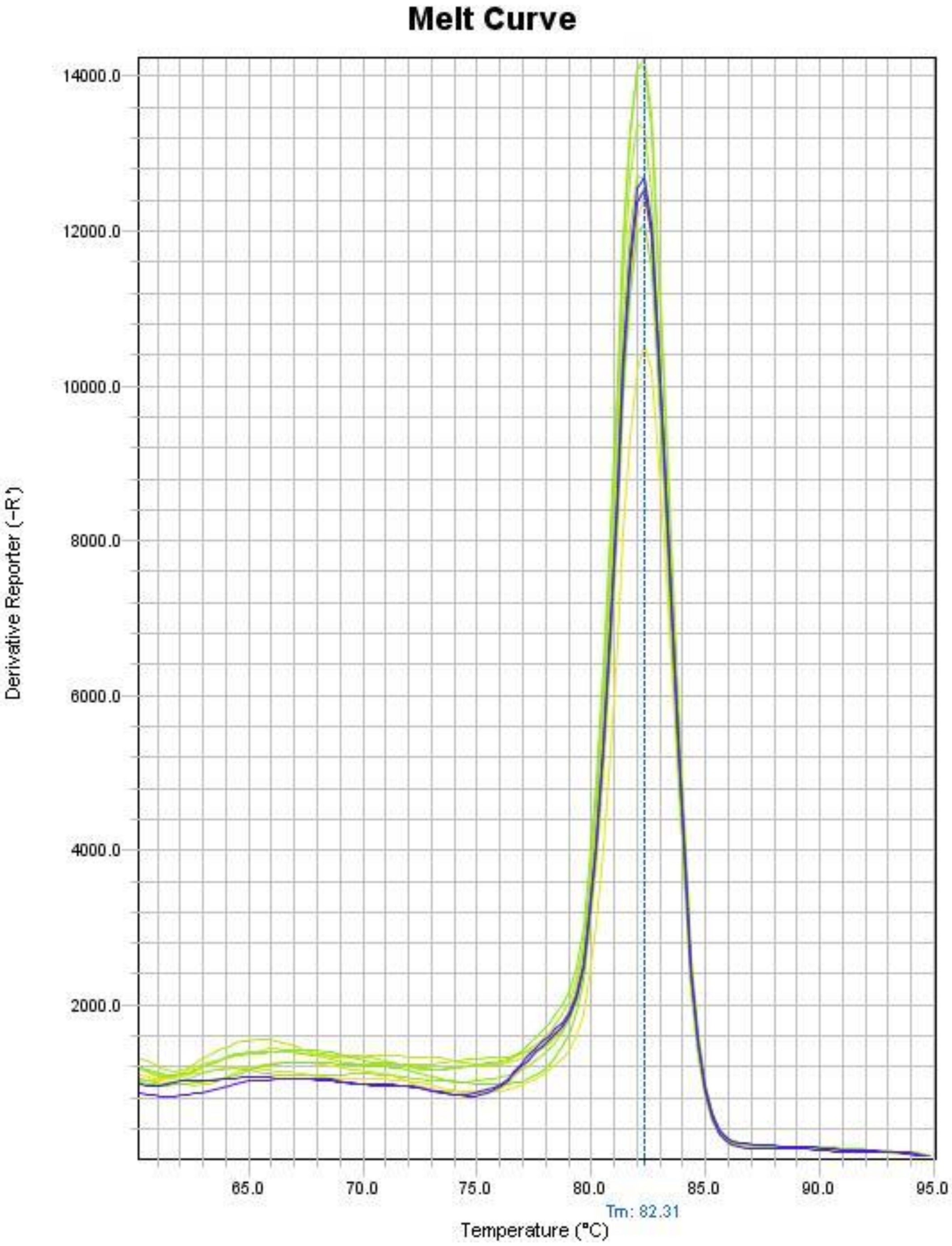

Melt Curve
14000.0
12000.0
10000.0
8000.0
6000.0
4000.0
2000.0
Derivative Reporter (-R')
65.0
70.0
75.0
80.0
85.0
90.0
95.0
Tm: 82.31
Temperature (°C)

Fig5_ATG7

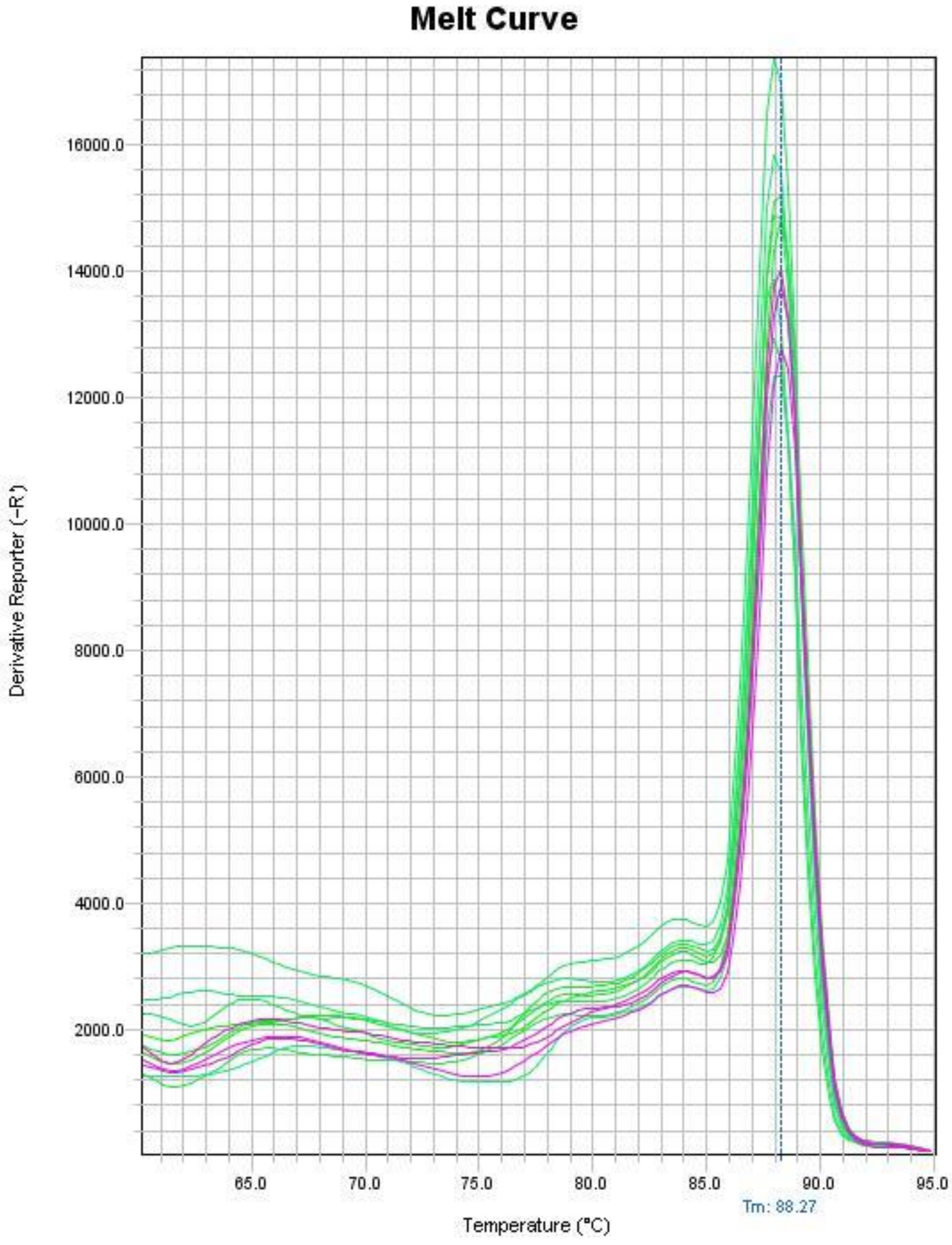

Melt Curve
16000.0
14000.0
12000.0
10000.0
8000.0
6000.0
4000.0
2000.0
Derivative Reporter (-R')
65.0
70.0
75.0
80.0
85.0
90.0
95.0
Tm: 88.27
Temperature (°C)

Fig5_ATG12

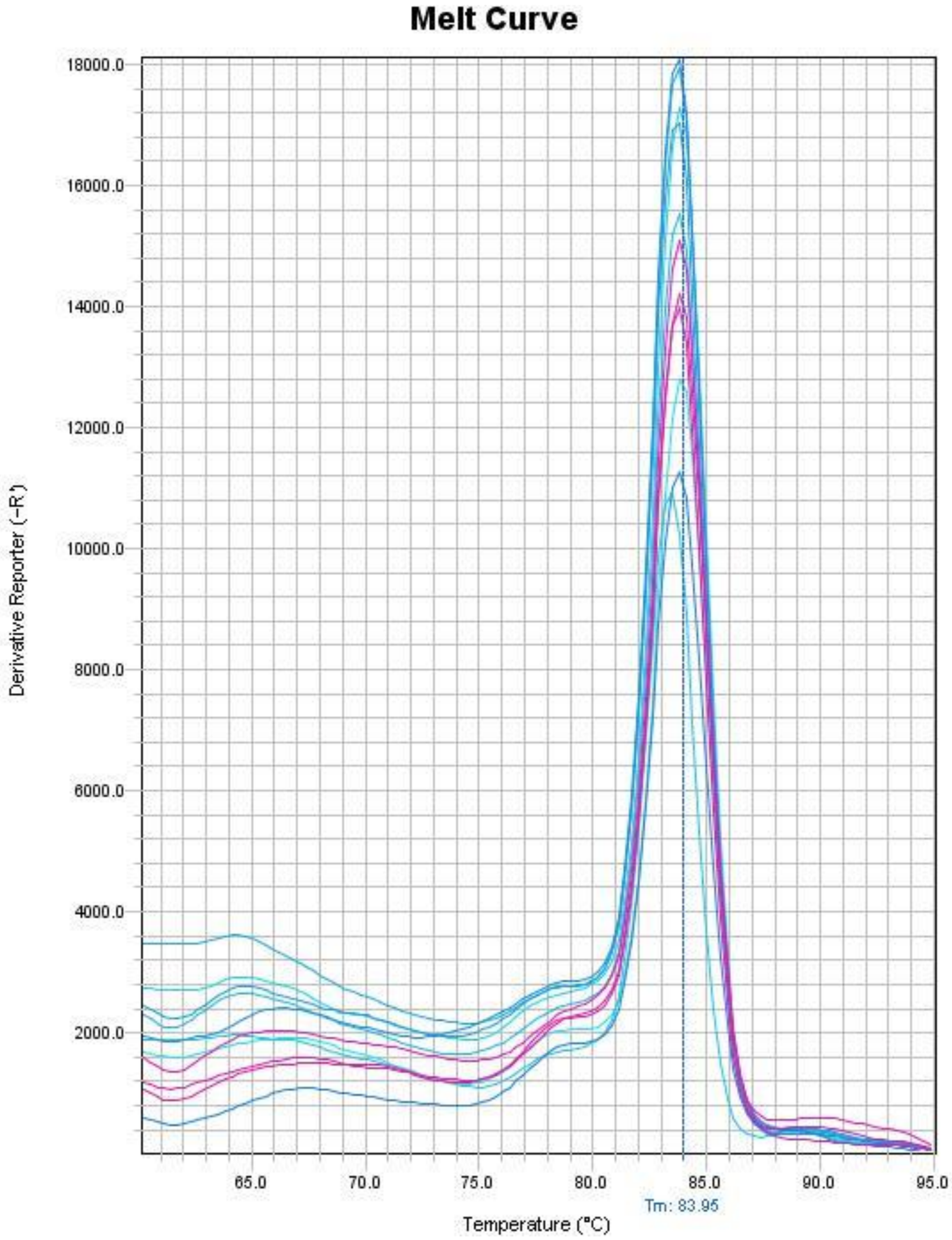
Melt Curve
18000.0
16000.0
14000.0
12000.0
10000.0
8000.0
6000.0
4000.0
2000.0
Derivative Reporter (-R')
65.0
70.0
75.0
80.0
85.0
90.0
95.0
Tm: 83.95
Temperature (°C)
