# Supplementary material for: Fermented mulberry (Morus alba) leaves suppress high fat diet-induced hepatic steatosis through amelioration of the inflammatory response and autophagy pathway
Source: BMC Complement Med Ther. 2020 Sep 18;20:283. doi: 10.1186/s12906-020-03076-2 (PMC7501671; doi:10.1186/s12906-020-03076-2)
Supplement: Supplementary file 2 — Additional file 2. [file 12906_2020_3076_MOESM2_ESM.pdf]

Supporting Materials 1

Original western blot image for p-JNK proteins in Fig. 3

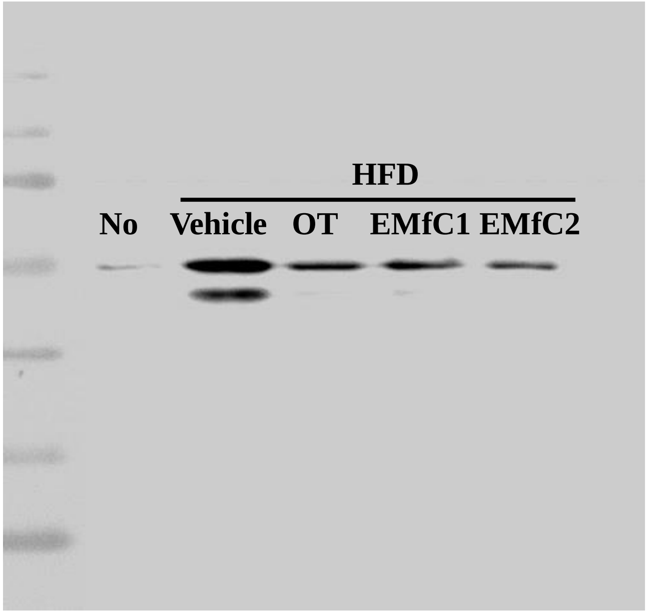

Expression of p-JNK in HFD+EMfC treated mice liver. Briefly, total lysates of liver were prepared using homogenizer and separated in SDS-PAGE gel. The expression level of actin protein with liver homogenate transferred on the membrane was determined by HRP-conjugated anti-rabbit IgG antibody during Western blot analysis.

Supporting Materials 2
Original Western blot image for JNK proteins in Fig. 3

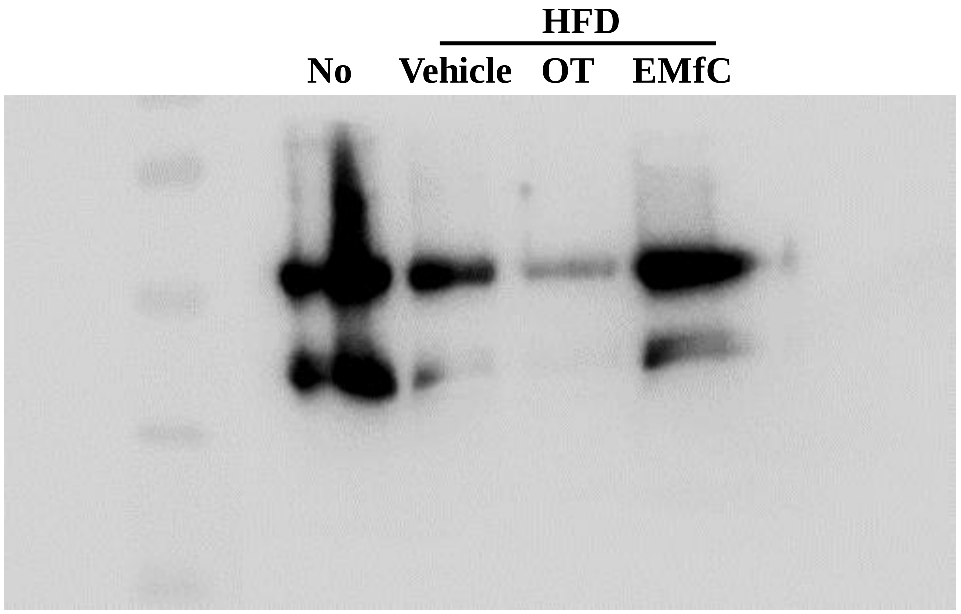

Expression of JNK in HFD+EMfC treated mice liver. Briefly, total lysates of liver were prepared using homogenizer and separated in SDS-PAGE gel. The expression level of actin protein with liver homogenate transferred on the membrane was determined by HRP-conjugated anti-rabbit IgG antibody during Western blot analysis.

Supporting Materials 3
Original western blot image for p-ERK proteins in Fig. 3

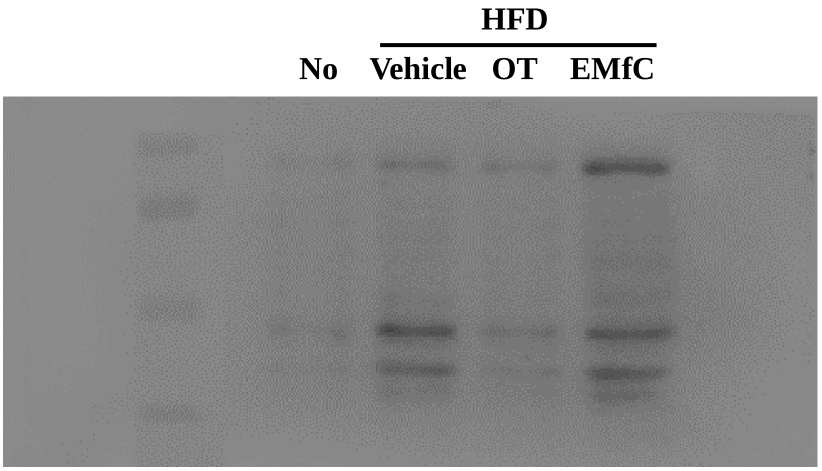

Expression of p-ERK in HFD+EMfC treated mice liver. Briefly, total lysates of liver were prepared using homogenizer and separated in SDS-PAGE gel. The expression level of actin protein with liver homogenate transferred on the membrane was determined by HRP-conjugated anti-rabbit IgG antibody during Western blot analysis.

Supporting Materials 4
Original western blot image for ERK proteins in Fig. 3

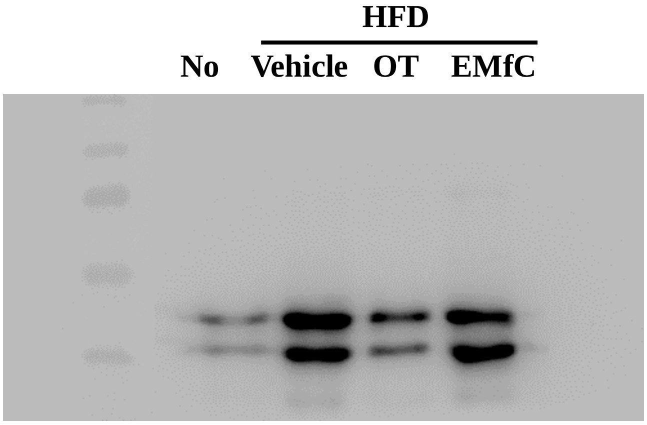

Expression of ERK in HFD+EMfC treated mice liver. Briefly, total lysates of liver were prepared using homogenizer and separated in SDS-PAGE gel. The expression level of actin protein with liver homogenate transferred on the membrane was determined by HRP-conjugated anti-rabbit IgG antibody during Western blot analysis.

Supporting Materials 5
Original western blot image for p-p38 proteins in Fig. 3

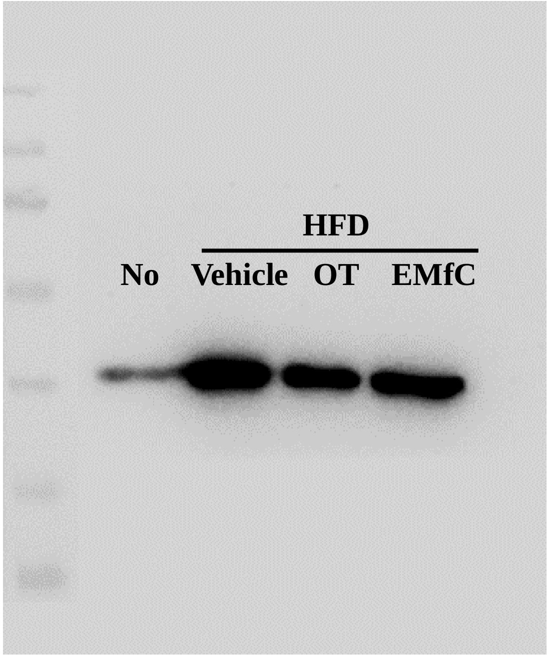

Expression of p-p38 in HFD+EMfC treated mice liver. Briefly, total lysates of liver were prepared using homogenizer and separated in SDS-PAGE gel. The expression level of actin protein with liver homogenate transferred on the membrane was determined by HRP-conjugated anti-rabbit IgG antibody during Western blot analysis.

Supporting Materials 6
Original western blot image for p38 proteins in Fig. 3

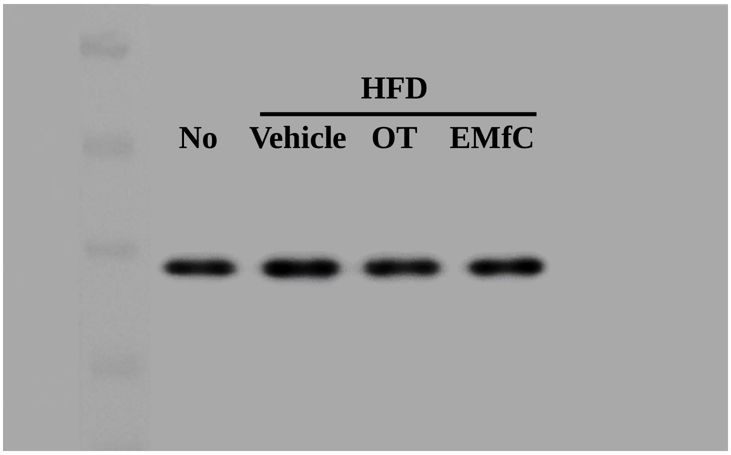

Expression of p38 in HFD+EMfC treated mice liver. Briefly, total lysates of liver were prepared using homogenizer and separated in SDS-PAGE gel. The expression level of actin protein with liver homogenate transferred on the membrane was determined by HRP-conjugated anti-rabbit IgG antibody during Western blot analysis.

Supporting Materials 7
Original western blot image for β-actin proteins in Fig. 3

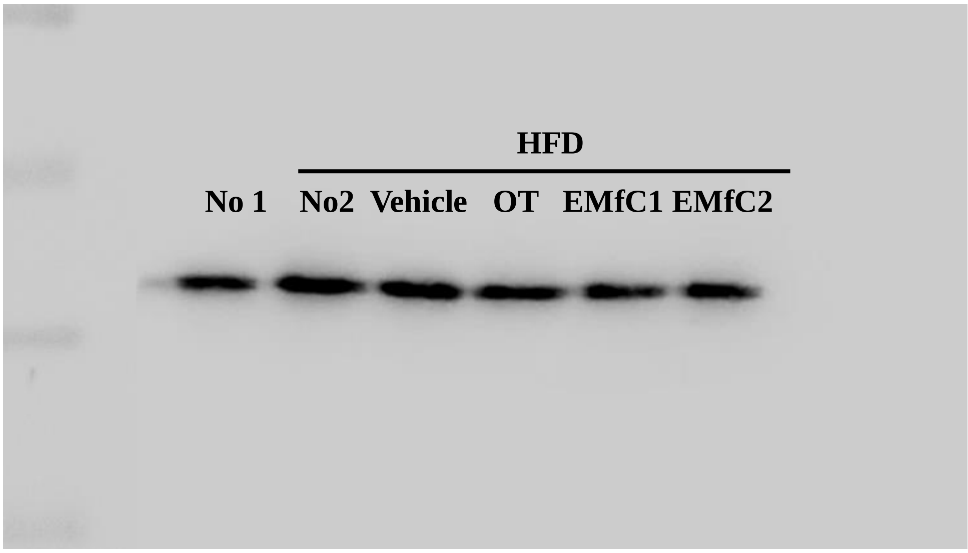

Expression of β-actin in HFD+EMfC treated mice liver. Briefly, total lysates of liver were prepared using homogenizer and separated in SDS-PAGE gel. The expression level of actin protein with liver homogenate transferred on the membrane was determined by HRP-conjugated anti-rabbit IgG antibody during Western blot analysis.

Supporting Materials 7
Original western blot image for p-PI3K proteins in Fig. 5

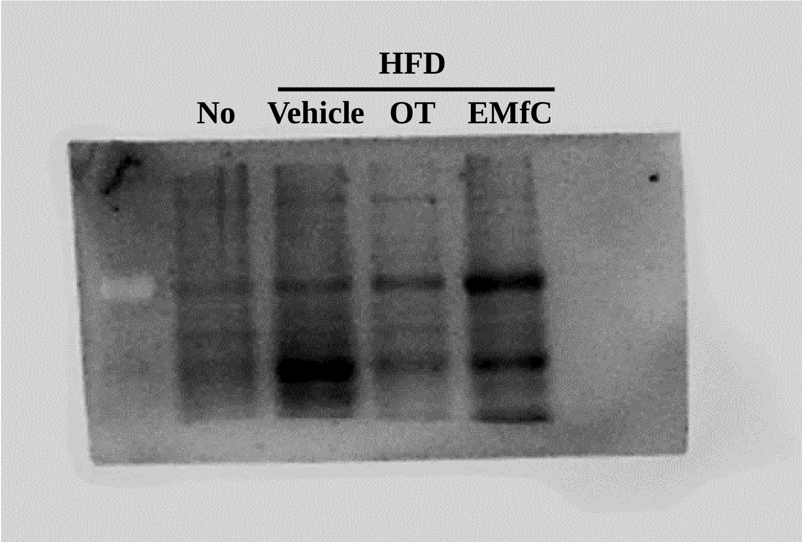

Expression of p-PI3K in HFD+EMfC treated mice liver. Briefly, total lysates of liver were prepared using homogenizer and separated in SDS-PAGE gel. The expression level of actin protein with liver homogenate transferred on the membrane was determined by HRP-conjugated anti-rabbit IgG antibody during Western blot analysis.

Supporting Materials 9
Original western blot image for PI3K proteins in Fig. 5

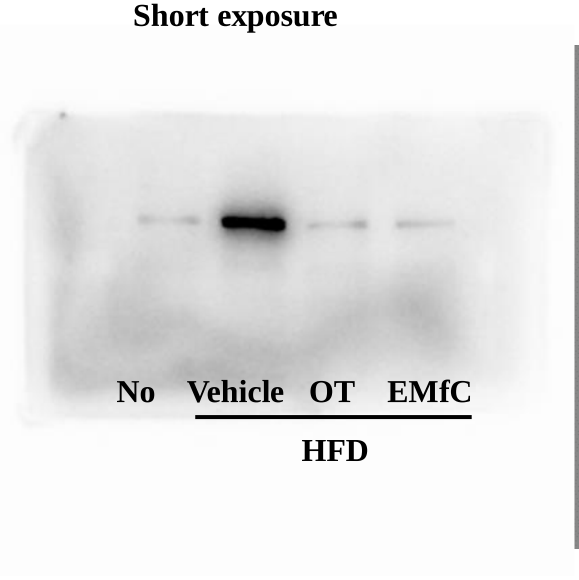

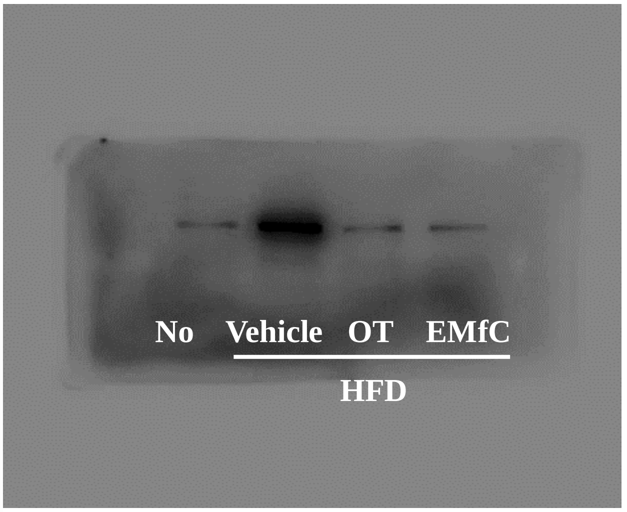

Expression of PI3K in HFD+EMfC treated mice liver. Briefly, total lysates of liver were prepared using homogenizer and separated in SDS-PAGE gel. The expression level of actin protein with liver homogenate transferred on the membrane was determined by HRP-conjugated anti-rabbit IgG antibody during Western blot analysis.

Supporting Materials 10
Original western blot image for p-AKT proteins in Fig. 5

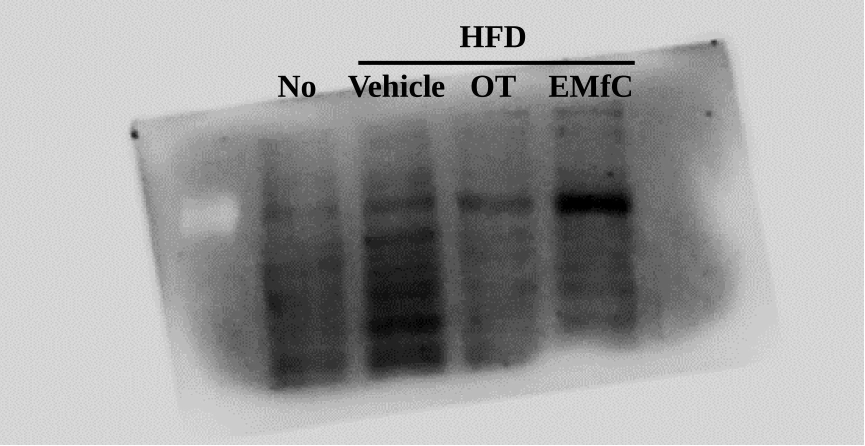

Expression of p-AKT in HFD+EMfC treated mice liver. Briefly, total lysates of liver were prepared using homogenizer and separated in SDS-PAGE gel. The expression level of actin protein with liver homogenate transferred on the membrane was determined by HRP-conjugated anti-rabbit IgG antibody during Western blot analysis.

Supporting Materials 11
Original western blot image for AKT proteins in Fig. 5

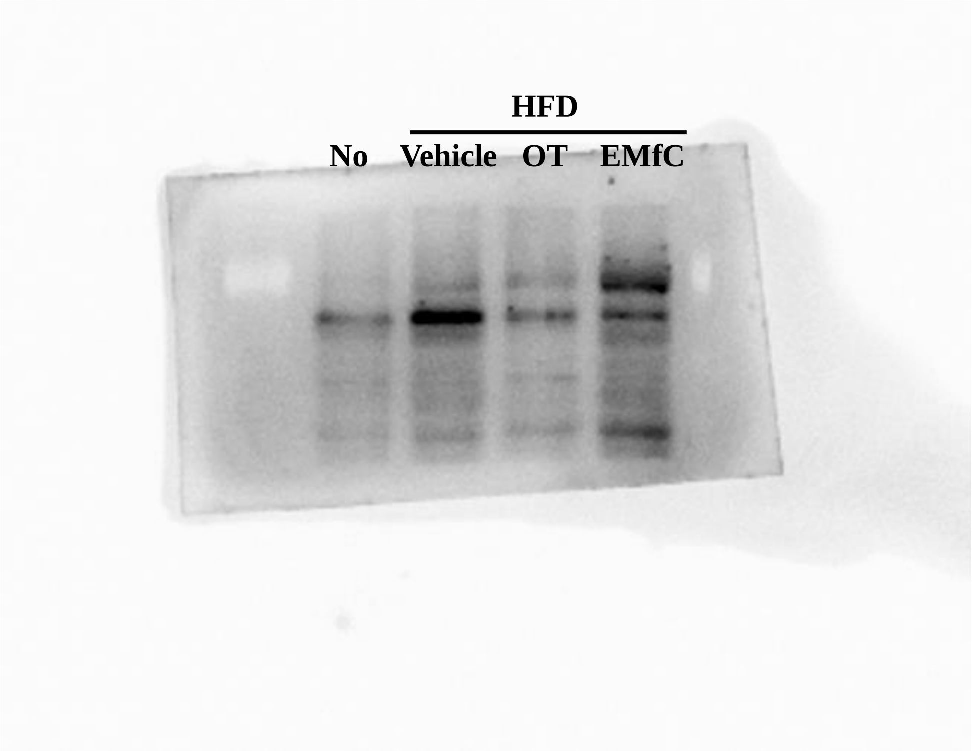

Expression of AKT in HFD+EMfC treated mice liver. Briefly, total lysates of liver were prepared using homogenizer and separated in SDS-PAGE gel. The expression level of actin protein with liver homogenate transferred on the membrane was determined by HRP-conjugated anti-rabbit IgG antibody during Western blot analysis.

Supporting Materials 12

Original western blot image for p-mTOR proteins in Fig. 5

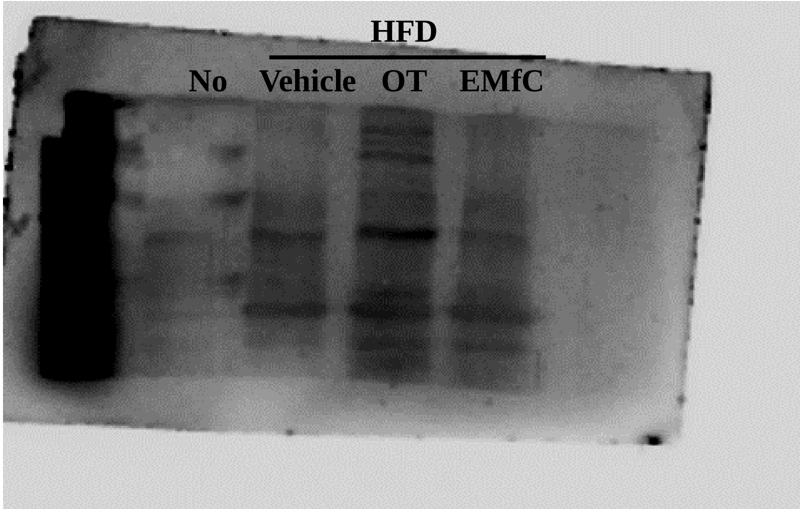

Expression of mTOR in HFD+EMfC treated mice liver. Briefly, total lysates of liver were prepared using homogenizer and separated in SDS-PAGE gel. The expression level of actin protein with liver homogenate transferred on the membrane was determined by HRP-conjugated anti-rabbit IgG antibody during Western blot analysis.

Supporting Materials 13

Original western blot image for mTOR proteins in Fig. 5

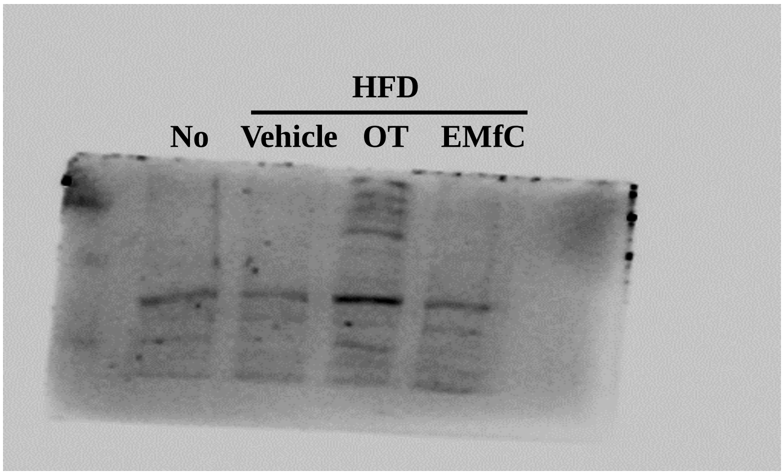

Expression of mTOR in HFD+EMfC treated mice liver. Briefly, total lysates of liver were prepared using homogenizer and separated in SDS-PAGE gel. The expression level of actin protein with liver homogenate transferred on the membrane was determined by HRP-conjugated anti-rabbit IgG antibody during Western blot analysis.

Supporting Materials 14
Original western blot image for β-actin proteins in Fig. 5

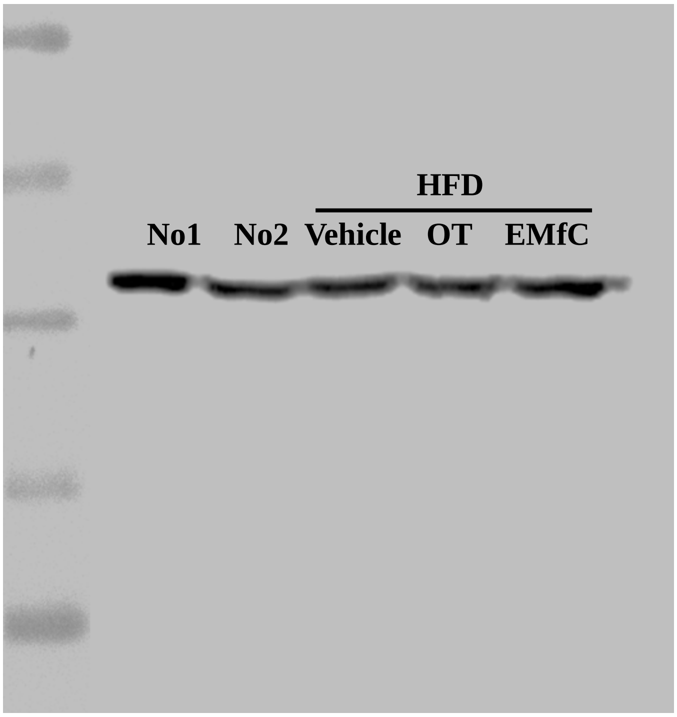

Expression of β-actin in HFD+EMfC treated mice liver. Briefly, total lysates of liver were prepared using homogenizer and separated in SDS-PAGE gel. The expression level of actin protein with liver homogenate transferred on the membrane was determined by HRP-conjugated anti-rabbit IgG antibody during Western blot analysis.

Supporting Materials 15
Original western blot image for Beclin proteins in Fig. 6

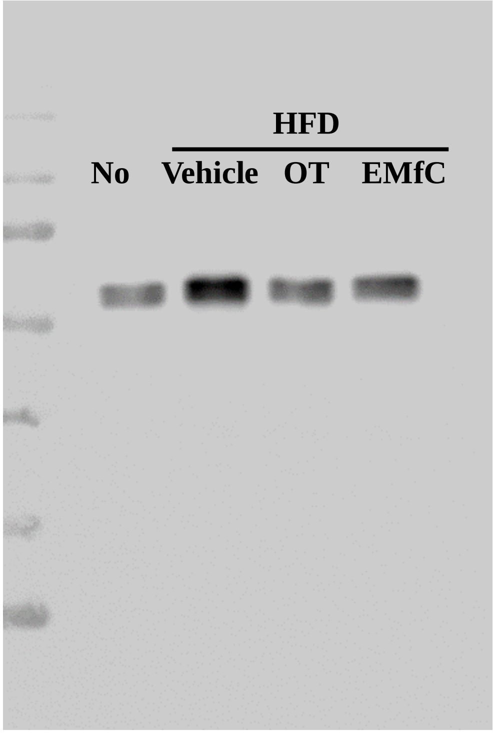

Expression of Beclin in HFD+EMfC treated mice liver. Briefly, total lysates of liver were prepared using homogenizer and separated in SDS-PAGE gel. The expression level of actin protein with liver homogenate transferred on the membrane was determined by HRP-conjugated anti-rabbit IgG antibody during Western blot analysis.

Supporting Materials 16
Original western blot image for LC3 proteins in Fig. 6

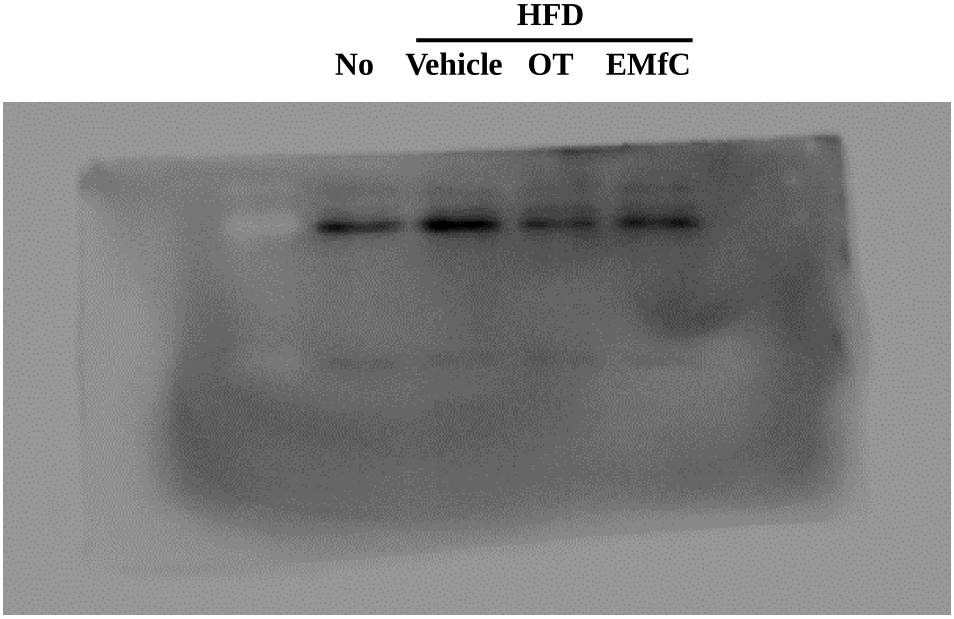

Expression of LC3 in HFD+EMfC treated mice liver. Briefly, total lysates of liver were prepared using homogenizer and separated in SDS-PAGE gel. The expression level of actin protein with liver homogenate transferred on the membrane was determined by HRP-conjugated anti-rabbit IgG antibody during Western blot analysis.

Supporting Materials 17

Original western blot image for β-actin proteins in Fig. 6

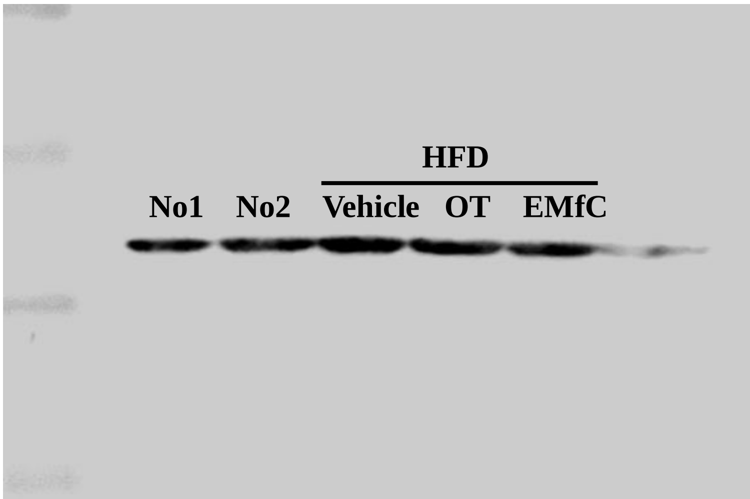

Expression of β-actin in HFD+EMfC treated mice liver. Briefly, total lysates of liver were prepared using homogenizer and separated in SDS-PAGE gel. The expression level of actin protein with liver homogenate transferred on the membrane was determined by HRP-conjugated anti-rabbit IgG antibody during Western blot analysis.
